# Quantitative Analysis of Glycerol Accumulation, Glycolysis and Growth under Hyper Osmotic Stress

**DOI:** 10.1371/journal.pcbi.1003084

**Published:** 2013-06-06

**Authors:** Elzbieta Petelenz-Kurdziel, Clemens Kuehn, Bodil Nordlander, Dagmara Klein, Kuk-Ki Hong, Therese Jacobson, Peter Dahl, Jörg Schaber, Jens Nielsen, Stefan Hohmann, Edda Klipp

**Affiliations:** 1 Department of Chemistry and Molecular Biology/Microbiology, University of Gothenburg, Göteborg, Sweden; 2 Theoretical Biophysics, Humboldt-Universität Berlin, Berlin, Germany; 3 Systems Biology Group, Department of Chemical and Biological Engineering, Chalmers University of Technology, Kemivägen, Göteborg, Sweden; 4 Institute for Experimental Internal Medicine, Medical Faculty, Otto von Guericke University, Magdeburg, Germany; Radhakrishnan Mahadevan, University of Toronto, Canada

## Abstract

We provide an integrated dynamic view on a eukaryotic osmolyte system, linking signaling with regulation of gene expression, metabolic control and growth. Adaptation to osmotic changes enables cells to adjust cellular activity and turgor pressure to an altered environment. The yeast *Saccharomyces cerevisiae* adapts to hyperosmotic stress by activating the HOG signaling cascade, which controls glycerol accumulation. The Hog1 kinase stimulates transcription of genes encoding enzymes required for glycerol production (Gpd1, Gpp2) and glycerol import (Stl1) and activates a regulatory enzyme in glycolysis (Pfk26/27). In addition, glycerol outflow is prevented by closure of the Fps1 glycerol facilitator. In order to better understand the contributions to glycerol accumulation of these different mechanisms and how redox and energy metabolism as well as biomass production are maintained under such conditions we collected an extensive dataset. Over a period of 180 min after hyperosmotic shock we monitored in wild type and different mutant cells the concentrations of key metabolites and proteins relevant for osmoadaptation. The dataset was used to parameterize an ODE model that reproduces the generated data very well. A detailed computational analysis using time-dependent response coefficients showed that Pfk26/27 contributes to rerouting glycolytic flux towards lower glycolysis. The transient growth arrest following hyperosmotic shock further adds to redirecting almost all glycolytic flux from biomass towards glycerol production. Osmoadaptation is robust to loss of individual adaptation pathways because of the existence and upregulation of alternative routes of glycerol accumulation. For instance, the Stl1 glycerol importer contributes to glycerol accumulation in a mutant with diminished glycerol production capacity. In addition, our observations suggest a role for trehalose accumulation in osmoadaptation and that Hog1 probably directly contributes to the regulation of the Fps1 glycerol facilitator. Taken together, we elucidated how different metabolic adaptation mechanisms cooperate and provide hypotheses for further experimental studies.

## Author Summary

Osmotic changes are common environmental challenges for cells, even in multi-cellular organisms, having led to sophisticated adaptation mechanisms. In order to adapt to hyperosmotic stress, yeast cells accumulate glycerol. This is achieved by short-term responses involving metabolic and transmembrane transport changes as well as long-term transcriptional responses. By integrating experimentation and simulation of a mathematical model we resolve the quantitative and temporal characteristics of different processes contributing to glycerol accumulation. We show that osmoadaptation prioritizes the redox and energy balance in glycolysis while rerouting flux from biomass to glycerol production. We further show that the glycerol accumulation network provides osmoadaptation with robustness by compensating for the loss of certain nodes and with the flexibility necessary for responding to different stress situations. Finally we provide novel insight into the roles of transport processes in glycerol accumulation and evidence that trehalose may play a role in yeast osmoadaptation. The present work provides for the first time an integrated dynamic view on a eukaryotic osmolyte system and links signaling with regulation of gene expression and metabolic control.

## Introduction

Upon increase in external osmolarity, cells first shrink and subsequently recover volume by accumulating compatible solutes [1]. Different processes contribute to adaptation, establishing negative feedback loops for the regulation of osmotic pressure, cell volume, and turgor [2]–[5]. The yeast *Saccharomyces cerevisiae* employs glycerol as compatible solute for osmo-regulation. Upon hyperosmotic shock the High Osmolarity Glycerol (HOG) pathway is activated, resulting in phosphorylation of the stress-activated protein (SAP) kinase Hog1. Phosphorylated Hog1 stimulates expression of genes encoding enzymes involved in glycerol production and uptake. Hyperosmotic stress also leads to rapid closure of the glycerol facilitator Fps1 preventing glycerol outflow. The contributions to glycerol accumulation of the different processes and regulatory mechanisms – including central metabolism - have not been systematically studied in a quantitative and time-resolved manner.

**Figure 1:**
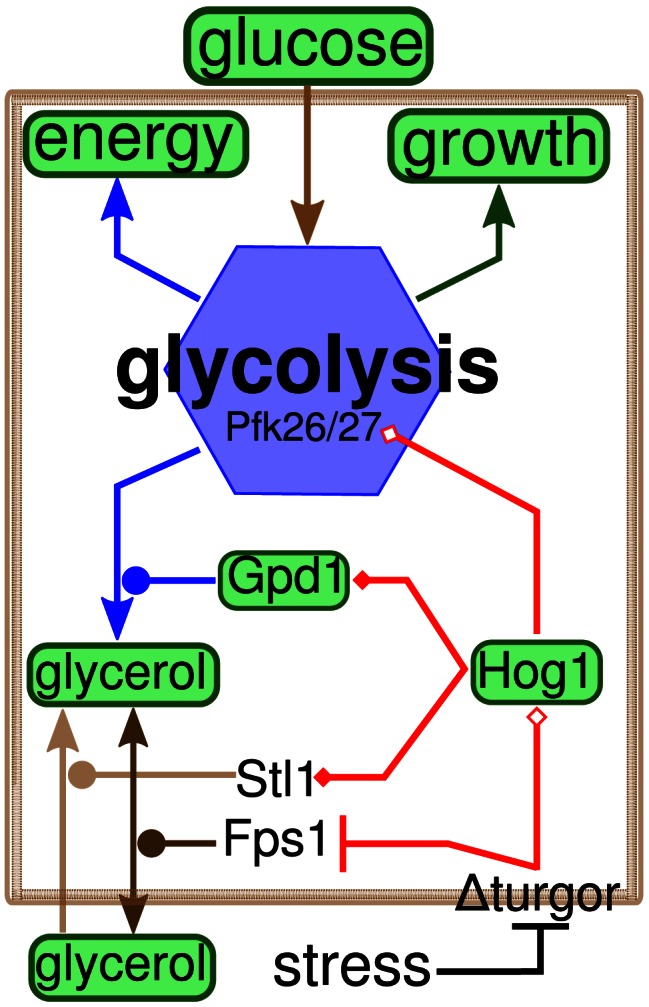
Overview of osmoadaptation in *S. cerevisiae*. Arrows indicate mass flow, diamonds indicate positive regulation (empty: direct, filled: gene expression), circles indicate catalysis, bars indicate inhibition. Measured entities are highlighted green (for a complete list of experiments refer to SI). Processes are colored according to the different modules (transport: brown, glycolysis: blue, growth: green, adaptation: red).

In the absence of hyperosmotic stress, glycerol production is required for maintaining the redox balance [1], [6] and excess glycerol leaks out freely through the glycerol facilitator Fps1 [7], [8]. The glycerol production pathway starts with the reduction of the glycolytic intermediate di-hydroxyl-acetone phosphate (DHAP) to glycerol-3-phosphate (G3P) catalyzed by the NAD^+^-dependent glycerol-3-phospate dehydrogenase. This enzyme is encoded by two isogenes, where osmostress controls expression of *GPD1* and cellular redox potential controls expression of *GPD2* [6], [9]–[11]. Stimulated expression of *GPD1* [12], [13] enhances glycerol production under hyperosmotic conditions. G3P is transformed to glycerol by the G3P phosphatases Gpp1/Rhr2 and Gpp2/Hor2 [6], [9], [10], [14]–[16]. Hog1 may also control the amount of DHAP available for glycerol production: it appears that Hog1 stimulates the 6-phosphofructo-2-kinase Pfk26, which, together with its isoform Pfk27, produces fructose-2,6-diphosphate (F26DP), an allosteric activator of the glycolytic enzyme phosphofructokinase (Pfk1) [17]. Glycerol transmembrane transport is facilitated by the aquaglyceroporin Fps1. Its rapid closure upon osmostress prevents glycerol outflow [18]. Active glycerol uptake is mediated by the glycerol-proton symporter Stl1, whose expression is strongly up-regulated upon osmoshock [19], [20] and down-regulated by glucose repression. Fig. 1 presents an overview of the known mechanisms involved in osmoadaptation.

Genetic tools to study osmoadaptation include a range of knockout mutants and several strains with alterations in regulatory nodes. The strain *FPS1-Δ1* contains a mutation that prevents the stress dependent closure of Fps1; this strain produces glycerol but loses it by diffusion through Fps1 [8]. *HOG1-att* contains a tag that tethers Hog1 to the plasma membrane. Such cells are osmo-resistant but do not mount a Hog1-dependent gene expression response [21].

In this study we integrate analysis of five distinct control mechanisms for glycerol accumulation and their link to glycolysis: (i) regulation of *GPD1* expression by nuclear Hog1, (ii) activation of Pfk26 by cytosolic Hog1, (iii) regulation of glycerol transport through Fps1, (iv) volume and associated concentration changes of all cellular compounds, and (v) metabolic reconfiguration resulting in rerouting of fluxes. To study the interplay between these mechanisms, we employed an iterative approach of modeling and experimentation. This model is based on a far more extensive dataset than several previous models including experimental data on a range of metabolites as well as the Hog1 and Gpd1 proteins in wild type and different mutants. The interaction between the different glycerol accumulation processes can be considered as a network in which each node represents one means of control. We tested the robustness of this network to genetic perturbations by model simulation and experimentation.

This work for the first time provides an overview of the dynamics of a eukaryotic osmolyte system, integrating biophysical changes with signaling, control of gene expression, and regulation of metabolic flux. Specifically, we provide evidence that (i) rerouting of glycolytic flux from growth to glycerol production is a major effector of osmoadaptation, (ii) different glycerol accumulation control mechanisms can (partly) compensate for each other, providing robustness and flexibility, (iii) long-term adaptation may involve other osmolytes than glycerol and (iv) Fps1-mediated glycerol efflux is down-regulated by active Hog1.

## Results

### Model

Based on previous work [22], pilot experiments, and a previous model [2] we constructed a refined mathematical model (Supplementary Information (SI) and Fig. S10). We included carbon flux to biomass, the influence of Hog1 on glycolytic flux *via* Pfk26/27, and Stl1-mediated glycerol uptake. This model comprises the following modules:

- *Biophysical changes*: changes in cell volume (basal solid volume, V_b_, and osmotically active volume, V_os_ [23], [24]), cell surface area, osmotic pressure, and turgor pressure (where changes depend on V_os_ [2] and external osmolarity).
- *Glycolysis module*: metabolic reactions from glucose uptake *via* phosphorylated intermediates to glycerol, trehalose, ethanol, and acetate. The module is based on models [2], [25] where reactions have been lumped to reduce the number of parameters (see Text S1 for details). The 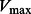 of the Pfk1 reaction is modulated by F26DP, which depends on the Hog1-regulated activity of Pfk26/27.
- *Transport module*: glucose, trehalose, ethanol, acetate transport, glycerol exchange *via* Fps1, and irreversible glycerol uptake *via* Stl1. We corrected rate laws of transport reactions for cell density increase in time-course experiments.
- *Biomass module*: biomass production is necessary for describing the interplay between osmotic regulation and glycolysis, but also to maintain correct carbon balance. Biomass is measured as cell number or cell density. Upon osmoshock, Hog1 arrests cell cycle progression [26], [27] and hence proliferation. Hog1-activity affects metabolism by altering expression of metabolic genes [20], [28]–[30]. A Hog1-dependent drop in growth rate is included in the model (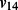).
- *Adaptation module*: Hog1 phosphorylation, *GPD1*, and *STL1* mRNA, translation to Gpd1 and Stl1, activation of Pfk26/27, and closure of Fps1. Hog1 activity is correlated with volume changes and turgor [24]. The Hog1 signaling system is described with only two reactions.

In order to ensure that the available data support the model, which covers different major processes, metabolism is highly condensed for this specific study, compared to metabolic reconstructions suitable for steady state analyses. For example, we refrained from including redox balance dynamics since they are affected by cellular changes beyond the scope of this model, such as detailed biosynthetic pathways (see Text S1). We do not assume a steady state for the entire model because we consider external metabolites and cell density. Although most intracellular concentrations can be considered constant before osmotic stress, the increasing external concentrations of ethanol, acetate and glycerol and the decreasing external glucose level together with the increase in OD slightly affect model dynamics. Therefore, the model is not in steady state in a strict sense.

Models for the different mutant strains are generated by implementing known effects of the respective genetic perturbation (see Text S1). Besides these modifications, all models use the same parameter set.

### Data set

**Figure 2:**
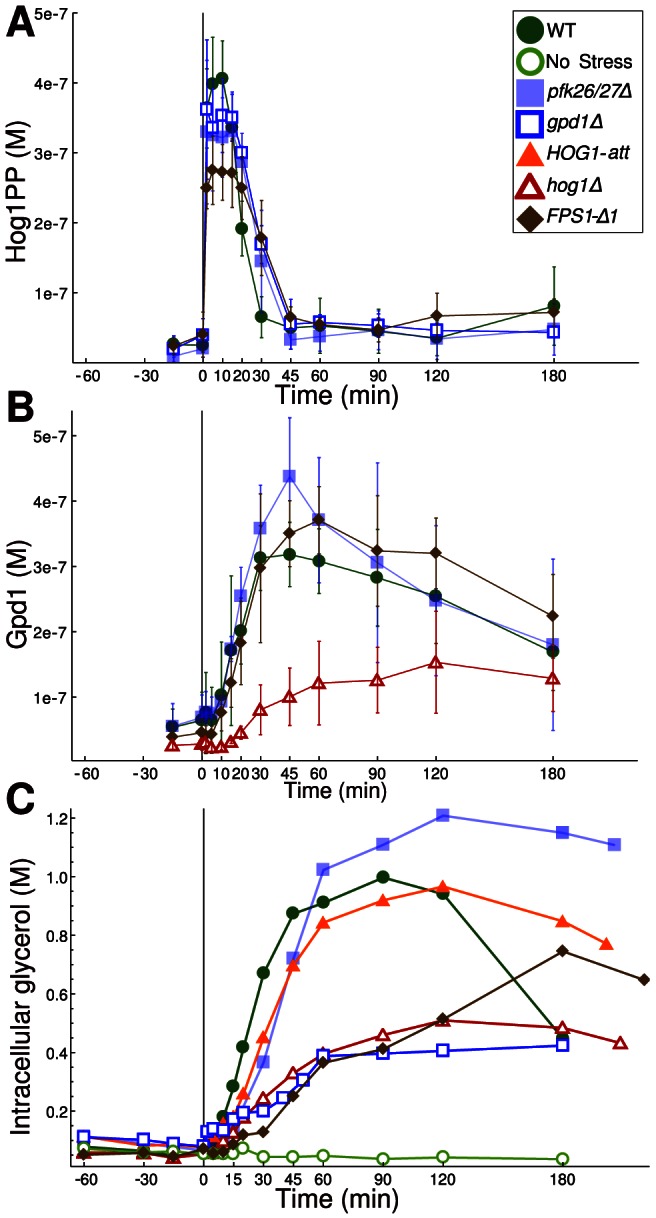
Time courses of (**A**) phosphorylated Hog1, (**B**) Gpd1, and (**C**) intracellular glycerol following hyperosmotic stress of 0.4 M NaCl at time point 0. The full dataset is provided in Datasets S1, S2, S3, S4, S5, S6, S7.

To estimate model parameters and determine time scales, we measured intracellular and extracellular concentrations of glycerol, glucose, ethanol, acetate, and trehalose over 240 min for wild type and mutants lacking particular adaptation mechanisms (*hog1Δ*, *gpd1Δ*, *pfk26Δ*, *pfk27Δ*, *pfk26/27Δ*) or with altered regulation nodes (*HOG1-att*, *FPS1-Δ1*) (Fig. 2, SI). While external glucose is consumed, glycerol, ethanol, and acetate as well OD_600_ and cell numbers (SI, Fig. S7) increased over time. Internal glycerol (Fig. 2C) accumulates transiently, while trehalose shows two peaks at 45 min and 180 min (Fig. S7G). We also measured *GPD1* mRNA concentration, Gpd1 concentration, and Hog1 phosphorylation (Fig. 2A–B, Fig. S13B). The following characteristics were noted.

- In the *gpd1Δ* mutant, the extracellular glycerol decreases, while the *FPS1-Δ1* strain excretes large amounts of glycerol (Fig. S8).
- Intracellular glycerol (Fig. 2C) builds up with a delay in the *FPS1-Δ1* and *hog1Δ* mutants. In the *pfk26/27Δ* and *Hog1-att* [21] strains, intracellular glycerol remains high over the entire period.
- The *pfk26/27Δ* mutant displays slower glucose consumption (Fig. S7B).
- Trehalose dynamics at 180 min indicate that it may accumulate further in wild type; *pfk26/27Δ* and *HOG1-att* strains do not display a late increase of trehalose (Fig. S7G).
- Hog1 phosphorylation (Fig. 2A) is slightly prolonged in cells lacking Gpd1 or Pfk26/27 or expressing constitutively open Fps1.
- The lowest Gpd1 levels are observed for the *hog1Δ* mutant (Fig. 2B).

### Simulation of osmoadaptation

The model was fitted to experimental data (Figs. S12, S13, S14). In general, model fitting can follow two different paradigms: (i) either fit the model to part of the data and use the rest of the data to test model predictions or (ii) use all data for fitting to obtain the model best describing the observations. Since our quantitative time-resolved data of various compounds for wild type and different mutants comprehensively elucidates the potential network dynamics, we decided to eventually use all data from all yeast strains and conditions to parameterize the final model presented here. To reproduce the Gpd1-mediated increase of glycerol production, dimerization of Gpd1, as reported for human Gpd [21], [31], [32], was implemented. We further implemented a partial Hog1-independent increase of *GPD1* mRNA to reproduce *hog1Δ* data, which is in accordance with experimental observations [21], [22]. To fit *gpd1*Δ mutant data, we incorporated a small osmo-dependent increase in *GPD2* transcription. Including regulation of biomass production [30] significantly improved model behavior (SI): A rapid drop in cell growth, dependent on stress and cell volume, is necessary to provide sufficient carbon for glycerol production and reproduce experimental data on cell density. To reproduce experimental data for intracellular glycerol in *hog1Δ* and *HOG1-att* strains, we included a negative regulation of open Fps1 by active Hog1.

### Model analysis and predictions

**Figure 3:**
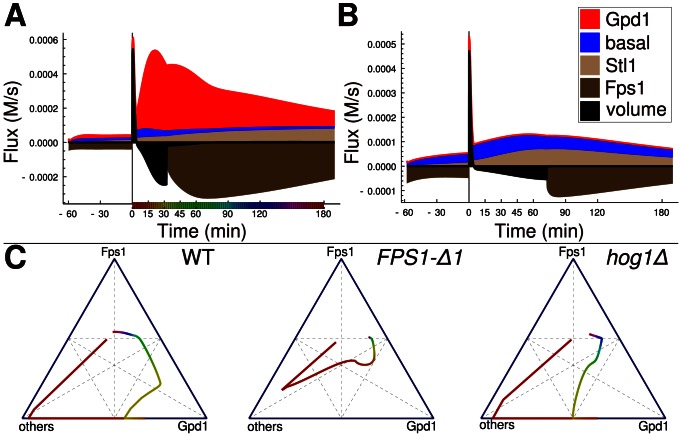
Contribution of glycerol accumulation mechanisms in different strains. (**A, B**) Absolute fluxes towards glycerol as well as relative contributions of specific mechanisms differ between wild type WT (**A**) and *gpd1Δ* (**B**). (**C**) Changes in relative contributions of Fps1, Gpd1, and other effects (basal glycerol production, uptake through Stl1, effects of volume change) over time are depicted for WT, *fps1*-*Δ1* and *hog1Δ*. Colors in (C) indicate time as shown on the x-axis in (A).

To characterize the contribution of different processes to osmoadaptation (Fig. 3), we plotted the absolute fluxes to and from glycerol for wild type and *gpd1Δ* over time (Fig. 3A and B) and relative contributions of fluxes in ternary plots (Fig. 3C). We distinguish Fps1-reliant, Gpd1-reliant, and other contributions (‘Others’) to net glycerol flux in each strain. For wild type, immediate adaptation is influenced mainly by changes in volume (part of ‘Others’) and closure of Fps1. Within approximately 15 min after stress, glycerol production becomes the predominant contribution. After 30 min, Fps1 reopens and glycerol levels decrease. Open Fps1 in *FPS1-Δ1* results in constitutive glycerol efflux. Although the model underestimates the glycerol production for this mutant, it clearly shows that the distribution of fluxes remains at a state of sustained glycerol production. Sustained glycerol production occurs also in the *hog1Δ* mutant, but due to reduced Hog1-dependent *GPD1* transcription its level remains low until 60 min after stress. Subsequently, the system moves to a state similar to that observed for the *FPS1-Δ1* strain.

**Figure 4:**
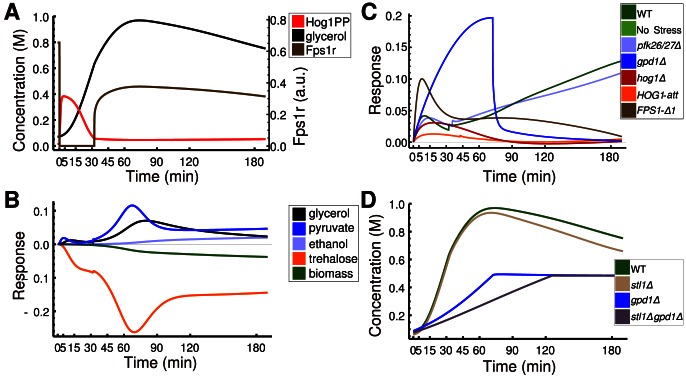
Model analysis with time-dependent response coefficients. **A**: Model simulation for phosphorylated Hog1, intracellular glycerol, and abundance of open Fps1. **B**: Effect of small changes in Pfk26/27 activation on different model variables as expressed by normalized response coefficient indicates that Pfk26/27 contributes to a rerouting of flux towards lower glycolysis. **C**: Response of intracellular glycerol concentration to perturbations in Stl1 gene expression as measured by normalized response coefficients in different strains indicates a specific time- and context-dependent role of Stl1in osmoadaptation. **D**: Simulation of genetic perturbations of Stl1 results in time courses as expected from C: in wild type, deletion of STL1 affects intracellular glycerol levels only at later time points. In *gpd1Δ* background, the effect of additional deletion of STL1 is early and transient.

Although the relative composition of fluxes approaches pre-stress values in wild-type, we do not observe perfect adaptation [33] (Fig. 4). For a more comprehensive analysis, the complete state of cells has to be considered, including changes in reactions or pathways indirectly affecting glycerol concentrations, e.g. changes in biomass production. To do so, we employed scaled time-dependent response coefficients (RCs), 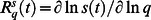 for a compound concentration 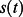 and a parameter *q* [34]. RCs express the relative change in 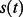 given a small change in *q*, serving as quantitative measure for the effect of a parameter perturbation on a time course taking all direct and indirect effects into account. Positive or negative values indicate that the time course increases or decreases upon parameter increase, respectively. Scaled RCs (Fig. 4B–C) show that regulation of Pfk26/27 does not have a pronounced effect on intracellular glycerol (Fig. 4B), which is in accordance with experimental data for *pfk26/27Δ* (Fig. 2C). Additionally, other reactions downstream of Pfk1 are positively affected while glycolytic reactions upstream of Pfk1 (trehalose and biomass production) are reduced. This indicates that Pfk26/27 might be part of a rerouting mechanism of metabolic flux ensuring that the influx to each branch of glycolysis is adjusted to demands and that ATP-production downstream of pyruvate is maintained during osmoadaptation.

We use RCs to compare the role of Stl1 in different experiments (Fig. 4C): apparently the contribution of Stl1, though negligible in most strains until 90 min after stress, is significant in *gpd1Δ*. *In silico* predictions show an early decrease in intracellular glycerol accumulation in the *gpd1Δ stl1Δ* double mutant compared with *gpd1Δ* but a late decrease in the *stl1Δ* mutant compared with wild type (Fig. 4D). The different roles of Stl1 in wild type and *gpd1Δ* cells highlight context specificity in osmoadaptation.

**Figure 5:**
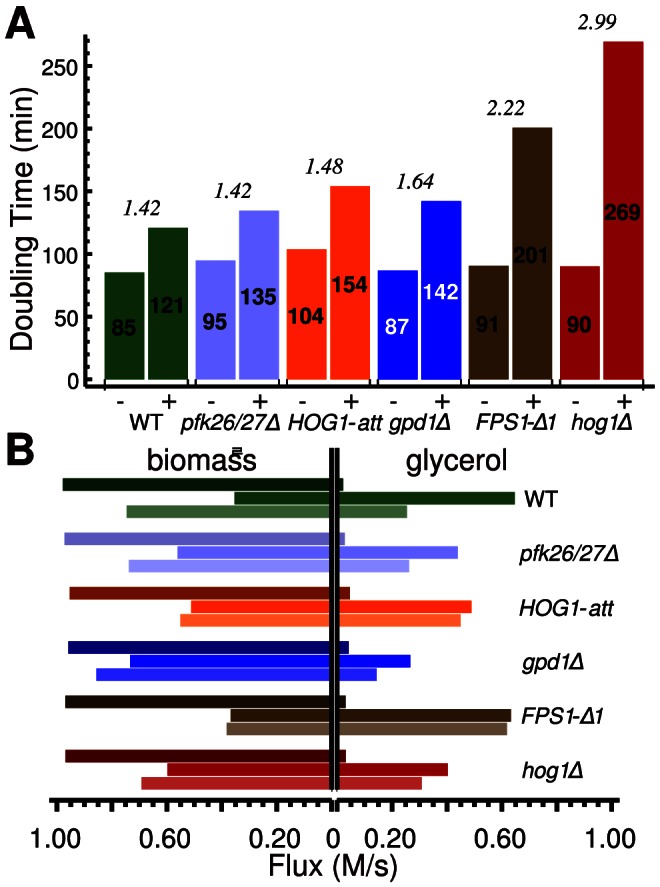
Effect of salt stress on growth rate. **A**: *In vivo* doubling times (−: before, +: after addition of 0.4 M NaCl) strongly differ between strains. **B**: Model simulations of the flux towards biomass production (left) and glycerol production (right) in the different strains at 0, 20, and 90 minutes after osmotic upshift to 0.4 M NaCl indicate a link between insufficient glycerol accumulation and a prolonged decrease in growth rate.

The regulation of biomass production significantly contributes to glycerol accumulation (Fig. 5, Fig. S15B). Rerouting of glycolytic flux can be assumed to result from Hog1-mediated cell-cycle arrest and glycolytic regulation. The cost of maintaining a certain cell volume by producing more glycerol is compensated by a decrease in growth rate. Changes in doubling times before and after stress are plotted in Fig. 5A. The observed decrease in growth rate is similar for wild type, *pfk26/27Δ*, and *HOG1-att* strains. This indicates that the main contribution of Hog1 activity to osmoadaptation is not the transcriptional activation of *GPD1*, which is absent in the *HOG1-att* strain. Instead, control of cytosolic or membrane-bound targets constitutes the main contributions of Hog1. A strong growth rate drop is observed in *FPS1-Δ1* and *hog1Δ*, while this drop is relatively low in the *gpd1Δ* mutant. This supports the idea that a Hog1-effect on Fps1 is mainly responsible for the prominent role of Hog1 in osmoadaptation. Fig. 5B shows model simulations of the relative carbon fluxes from glycolysis to glycerol or biomass production, respectively, for wild type and different mutants before 20 min and 90 min after stress induction, indicating a trade-off of cellular adaptation *versus* growth.

The model is fitted to data covering a period until 180 min after stimulation. However, cellular processes beyond the scope of the model may interfere with the adaptation process at later stages, hence model results and hypotheses derived from those may be less reliable beyond 120 min.

## Discussion

For the first time we present a data-driven analysis of the dynamic regulation of a eukaryotic osmolyte system mediating adaptation to hyperosmotic stress that integrates different regulatory layers. Our mathematical model, supported by a detailed dedicated dataset, provides novel insight into the quantitative contributions of regulatory processes underlying glycerol accumulation during the yeast osmostress response.

The main conclusions are:

- The Hog1 SAPK mediates regulation of glycolysis via indirect activation of the enzyme Pfk1 and increased expression of glycerol-producing enzymes.
- Pfk1 activation serves stabilization of glycolytic flux as much as increased glycerol accumulation.
- Glycerol accumulation occurs at the expense of biomass production.
- Glycerol accumulation is achieved by different fluxes comprising basal (Gpd2-dependent) and Hog1-regulated (Gpd1-dependent) production, glycerol exchange over the plasma membrane as well as concentration increase through volume loss; these fluxes contribute to varying extent during different periods of adaptation.

The parameter set was fitted to reproduce data from seven different strains and experimental observations on 14 of 29 model variables, providing the model with an unprecedented coverage [35]. This coverage was achieved employing coordinated model reduction and experimentation with the intention to maintain all regulatory nodes while lumping metabolites and proteins for which no experimental data was gathered.

Deleting individual nodes of the glycerol accumulation network (Figs. 1, S10) results in slightly prolonged HOG pathway activation. For *FPS1-Δ1*, this prolongation is less pronounced than in previous data [2], which is probably due to different copy numbers of the expression construct. In contrast to Dihazi et al. [17], we found that only the double deletion mutant, *pfk26Δpfk27Δ* shows prolonged Hog1 phosphorylation. The mutation causes a transient delay of glycerol accumulation (Fig. 2C).

We hypothesize that, like human Gpd1 [32], also yeast Gpd1 dimerizes since this assumption results in significantly better fits to experimental data.

It has been shown that Hog1 interacts with and controls Fps1 under certain conditions [36]–[38], although the exact mechanism of Hog1-dependent control of Fps1 remains elusive. Experimental observations (e.g. [8]), as well as the simulations shown here and elsewhere [5], are consistent with a Hog1-dependent down-regulation of glycerol flux through Fps1 under osmostress. In *hog1Δ* cells the glycerol efflux is elevated while membrane-attached Hog1 results in a very strict regulation.

In the *gpd1Δ* mutant the glycerol production rate is diminished. Our experimental data show that in this strain the extracellular glycerol concentration decreases over time, indicating glycerol uptake through the Stl1 glycerol-proton symporter. We determined the role of Stl1 in glycerol accumulation using time varying response coefficient analysis [34]. Our simulations indicate that the *stl1Δgpd1Δ* mutant adapts even worse than the *gpd1Δ* mutant and does not show a decrease in the extracellular glycerol concentration. This indicates that osmostress response can overrule glucose repression of Stl1, which is supported by experimental data from Ferreira et al [19] showing strongly stimulated *STL1* gene expression under osmostress in mutants unable to produce glycerol as well as growth phenotypes of such mutants lacking in addition Stl1.

The presented model underestimates the production of glycerol for *FPS1-Δ1*. This may have two reasons. First, *FPS1-Δ1* cells adapt to the imposed genetic perturbation prior to stress, which can, for example, lead to an up-regulation of glycerol production. Second, the proposed interaction of the modified protein with Hog1 or other potential regulators [38] may be perturbed. Thus, the model indicates that *FPS1-Δ1* has global effects beyond the expected de-regulation of glycerol efflux that need to be investigated further.

In adapted yeast cells the intracellular glycerol level diminishes. We observed an increase in intracellular trehalose concentrations coinciding with the decline of intracellular glycerol (Fig. S7G). In the *FPS1-Δ1* strain, which is unable to accumulate intracellular glycerol, the intracellular trehalose concentration is permanently high. Although the combination of high trehalose and glycerol concentrations has a synergistic osmoprotective effect [39], the observed trehalose concentrations are too small to significantly contribute to intracellular osmotic pressure. Hence, trehalose may have more specific roles in osmoadaptation by protecting proteins and membranes [40], [41].

We do not observe perfect adaptation in the sense defined previously [33]. Although cell volume returns to its original values, other variables remain perturbed in adapted cells at constant high external osmolarity, such as Gpd1, trehalose or glycerol concentration, and especially growth rate.

Glycolysis serves three major fluxes resulting in the production of ethanol plus acetate, biomass, and glycerol. Although active Hog1 leads to cell cycle arrest [26], [27], potentially decreasing carbon flux towards biomass, a role of glycolysis in the osmostress response has not been considered in detail. Data presented by Dihazi et al. [17] as well as recent gene expression data [42] indicate that glycolytic flux may play a role in osmoadaptation. Our preliminary theoretical work [22] indicated that Pfk26/Pfk27 participate in maintaining the flux towards pyruvate upon adaptation to hyper-osmotic conditions. Glucose consumption and ethanol production rates are similar in all strains studied here, regardless which node of the glycerol accumulation network was removed. The *pfk26/27Δ* strain is the only exception: the slightly lower ethanol production and glucose consumption observed in this mutant indicate that Pfk26/27 influence glycolysis rather than osmotic adaptation. We conclude that under hyper-osmotic stress part of the carbon flux, which is normally directed towards biomass production, is used for increasing glycerol production. One of the elements in this metabolic prioritization mechanism is Pfk26/27, although it does not seem to be the only factor. The ratio between the production of glycerol and pyruvate (resulting in the formation of ethanol and acetate) is crucial for a proper energy and redox balance. In all strains, the flux towards pyruvate remains unaffected by osmoadaptation while flux towards biomass decreases. Apparently, energy production and redox balance are maintained while growth is temporarily stopped for faster adaptation. This pattern is likely to hold for a wide variety of stresses and adaptation mechanisms.

The relative contribution of each individual glycerol accumulation mechanism depends on the environmental conditions and the physiological state of the cell (Fig. 3A). In their natural environment, cells have to cope with combinations of stresses demanding for dynamic modulation of response mechanisms. The different mechanisms contributing to osmoadaptation might make the process robust but also allow choosing the ‘cheapest’ adaptation strategy under different conditions.

Response coefficient analysis is an excellent tool to elucidate this kind of interdependences. We found that introducing an individual deletion into the glycerol accumulation network is compensated. Our experimental data show that the adaptation takes longer for single knock-out strains, compared to wild type. The activation of the remaining glycerol accumulation mechanisms is prolonged (active component of the compensation) and the remaining mechanisms become more important (passive compensation component).

Minimalistic models of biological processes, such as the osmoadaptation model presented by Mettetal et al. [3] are of great value for understanding general principles underlying a given process. With a sufficient degree of generalization, conclusions drawn from minimalistic models can be applicable for characterizing other cellular events. This approach neglects the individual mechanisms contributing to a cellular function. On the other extreme, the approach of hierarchical control analysis [43] measures the effects of changes on a cellular process in an enormous amount of detail. This approach is crucial for understanding the role of individual components and events. Our work attempts to bridge between the two paradigms, which can be viewed as an intermediate stage of different magnifications of the same picture.

The principles of osmoregulation are conserved from yeast to mammalian cells. The mammalian stress-activated protein kinase p38 is a homolog of Hog1. Like Hog1 it plays a critical role in mounting the adaptive response to stress by controlling metabolism, gene expression and cell cycle progression [44]. Hence, the approaches employed in the present work as well as the conclusions drawn may have consequences for studies on mammalian cells as well.

By integration of experimental data for different strains into a medium-size model and reliably estimating its parameters, we were able to achieve a better understanding of the contribution of individual players to a cellular response – osmoadaptation – in a quantitative and time-resolved manner. Moreover, our analysis has revealed the trade-off between growth control and glycerol accumulation in the adaptation process.

## Materials and Methods

### Experimental methods

#### Yeast strains and culturing

Strains used in this work originate from W303-1A (MATa leu2-3/112 ura3-1 trp1-1 his3-11/15 ade2-1 can1-100 GAL SUC2 mal0) [45]. The strains include: wild type, *gpd1Δ*, *pfk26Δpfk27Δ*, *fps1-Δ1* (kindly provided by Markus Tamás, University of Gothenburg), *stl1Δ*, *hog1Δ*, *HOG1-att* (kindly provided by Jeremy Thorner, University of California at Berkeley). A complete list of strains used in this study is presented in Table 1. Yeast cultures were grown until mid-exponential phase (OD_600_ = 0.7–1.0) in YPD medium (Yeast Peptone D-glucose; 1% yeast extract (Bacto), 2% peptone (Bacto), 2% glucose), then NaCl was added from a stock solution of 5 M in water to a final concentration of 0.4 M at t = 0 min.

**Table 1:** Yeast strains used in this study.

Strain name	Strain genotype	Origin
W303-1a	MATa leu23/112 ura31 trp11 his311/15 ade21 can1100 GAL SUC2	Thomas and Rothstein (1989) [45]
YMR84	W303-1A with gpd1Δ::URA3	Martijn Rep (Amsterdam)*
YSH1583	W303-1A with pfk26Δ::KanMX	This study
YSH1585	W303-1A with pfk27Δ::KanMX	This study
YSH1586	W303-1A with pfk26Δ::KanMX pfk27Δ::KanMX	This study
YMT101	W303-1A with fsp1Δ::LEU2 Ylp-URA3-fps1-Δ1	Tamas et al., (1999) [8]
YSH2293	W303-1A with stl1Δ::KanMX	This study
YSH444	W303-1A with hog1Δ::TRP1	Albertyn et al,. (1994) [12]
	W303-1A with hog1-att	This study

*Strain YMR84 was kindly provided by Martijn Rep (Amsterdam) and contains a replacement of the GPD1 upstream region (−883 to +91) by the URA3 gene. The strains was generated using a PCR approach and does not express the GPD1 gene product.

#### Western blot analysis

Samples of 1 ml were collected at the indicated time points, sedimented and frozen in liquid nitrogen after removing the supernatant. Proteins were extracted by boiling for 10 min in extraction buffer (100 mM Tris-HCl pH 6.8, 20% glycerol, 200 mM DTT, 4% SDS, 10 mM NaF, 0.1 mM Na3V04 (sodium orthovanadate), protease inhibitor (Complete EDTA-free Protease Inhibitor Cocktail tablets, Roche), and 20 mM mercapto-ethanol). The extracts were claried by centrifugation (13 000 rpm in 4°C for 10 min). For each sample 40 µg of protein was separated by electrophoresis on a 10% polyacrylamide gel (SDS-PAGE) and transferred (semi-dry) to a nitrocellulose membrane (Hybond-ECL, Amersham). Membranes were blocked with Odyssey Blocking Buffer (Li-Cor Biosciences) and incubated sequentially with primary antibodies: first primary antibody - phospho-p38 MAPK (Thr180/Tyr182) monoclonal rabbit antibody (Cell Signalling), 1∶1000 in Odyssey Blocking Buffer with TBST (1∶1000), overnight at 4°C; second primary antibody - yC20 total Hog1 polyclonal goat antibody (Santa Cruz Biotechnology Inc.), 1∶2000 in Odyssey Blocking Buffer with TBST (1∶1000), 1 h at room temperature, third primary antibody - rabbit polyclonal antisera Gpd1-A (Innovagen), 1∶2000 in Odyssey Blocking Buffer with TBST (1∶1000), 1 h at room temperature, and simultaneously with secondary antibodies: donkey anti-goat IR Dye 680, 1∶12 500 and donkey anti-rabbit IR Dye 800CW 1∶12500 (Li-Cor Biosciences), in Odyssey Blocking Buffer with TBST (1∶1000), for 45 min at room temperature.

The membranes were scanned using Odyssey Infrared Imaging System (Li-Cor Biosciences) and quantified using Multi Gauge 3.0 (FujiFilm) software.

#### Metabolite measurements

Samples of 1 ml were collected at the indicated time points. Three types of samples were collected for each time point: intracellular - centrifuged cell pellet without supernatant, extracellular - pure supernatant, removed from intracellular samples, total - 1 ml cells in medium, and frozen in liquid nitrogen. Extracellular samples did not require further processing; total extracts were boiled for 10 min and cleared by centrifugation, cell pellets were extracted with sterile water by boiling for 10 min and cleared by centrifugation. The concentrations of glucose, trehalose, glycerol, acetate, succinate, pyruvate and ethanol were measured by high performance liquid chromatography (DIONEX) with an Aminex HPX-87H ion exchange column (Bio-Rad, Hercules, USA). An isocratic condition was performed with 5 mM H2SO4 as mobile phase at flow rate of 0.6 ml/min and oven temperature of 65°C. Glucose, trehalose, glycerol and ethanol were quantified by a refraction index detector (Waters 410 Differential Refractometer Millipore, CA, USA) and acetate, succinate and pyruvate by ultraviolet-1 visible light absorbance detector (Waters 486 Tunable Absorbance Detector set at 210 nm, Millipore, CA, USA).

#### Data processing and additional data

Experimental data was processed to account for cell density increase and the reliability of HPLC measurements was assessed by comparison with enzyme assay quantification. For a full description of data processing and additional data, see Text S1.

### Mathematical modeling

System dynamics were described by ordinary differential equations (ODEs). A complete list of model equations and parameter estimation procedures is provided in SI. Time-dependent response coefficients have been calculated as described in [34]. Temporal simulations were performed with Mathematica7 (Wolfram Research. Mathematica edition: Version 7.0, 2008). Parameter estimation was done with PottersWheel [46] and SBML-PET [47].

## Supporting Information

Dataset S1 Raw and processed metabolite measurements for different strains, stress 0.4 M NaCl added at *t* = 0.
(XLS)

Dataset S2 Overview of experimental data as used in fitting.
(XLS)

Dataset S3 Comparison of intracellular trehalose measurements by HPLC and enzyme assay, stress 0.4 M NaCl added at *t* = 0.
(XLS)

Dataset S4 Western Blot data for different strains and stress strengths.
(XLS)

Dataset S5 Intracellular glycerol quantification by enzyme assay in different strains for different stresses added at *t* = 0.
(XLS)

Dataset S6 Intracellular trehalose quantification by enzyme assay in different strains, stress 0.4 M NaCl added at *t* = 0.
(XLS)

Dataset S7 Northern Blot results for CTT1, GRE2, STL1, 18S in different strains.
(XLS)

Figure S1 Cell density and optical density in stressed (0.4 M NaCl added at *t* = 0) and control experiments (A) and normalized to the values at *t* = 0 (B). Experimental setup as explained in main text.
(PDF)

Figure S2 Plot of OD versus cell density values as obtained from control and stressed time course experiments and the function for computing cell density from OD.
(PDF)

Figure S3 Measured (blue) and inferred (pink) intracellular glycerol for different experiments. Errors in the inferred values due to inconsistencies in measurements are visible in *FPS1-Δ1*, *hog1Δ* and WT1.
(PDF)

Figure S4 Comparison of glycerol time courses for WT1 and WT4. (A) intracellular glycerol, (B) extracellular glycerol.
(PDF)

Figure S5 Comparison of enzyme assay (blue) and HPLC (pink) intracellular glycerol quantifications. Underlying data is given in Supplemental Dataset S2.
(PDF)

Figure S6 Comparison of intracellular trehalose levels obtained with different methods. Enzyme assay data is in g D-glucose/l per µg protein/ml, HPLC data in mol/l is normalized to the enzyme assay value at *t* = 30. Underlying data is given in Supplemental Dataset S3.
(PDF)

Figure S7 Experimental data as used for model fitting. Measured entities are indicated on y-axes. Shown are representative experiments for each strain. Stress of 0.4 M NaCl is added at *t* = 0. A: intracellular glucose, B: extracellular glucose, C: Optical density, D: intracellular ethanol, E: extracellular ethanol, F: extracellular acetate, G: intracellular trehalose, H: extracellular trehalose, I: extracellular glycerol.
(PDF)

Figure S8 Experimental data for extracellular glycerol (A,B) and Gpd1 (C) following addition of 0.4 M NaCl at t = 0. Close inspection reveals a decrease of extracellular glycerol in *gpd1Δ* strain. The decrease intracellular glycerol in *gpd1Δ* is on a similar timescale as the transcriptionally regulated increase of Gpd1 in other strains, indicating that a transcriptionally regulated mechanism is responsible for this decrease as well.
(PDF)

Figure S9 Effects of *HOG1-att* (Hog1 attached to the plasma membrane) compared to wild-type. Under unstressed conditions, inactive Hog1 (dark red stars) is localized throughout the cell, residual active Hog1 (light red stars) is localized in the nucleus. Possible Hog1 interaction partners are depicted: Fps1 (brown triangles), Pfk26/27 (blue spirals) and genes (black waves). In wild-type, osmoadaptation leads to active Hog1 translocating to the nucleus to stimulate transcription. In *HOG1-att*, transcriptional regulation is abolished and cytosolic Hog1-concentration is reduced while possible interactions with membrane-bound proteins are increased.
(PDF)

Figure S10 Model topology in SBGN syntax. The different modules are color coded (red: adaptation, yellow: biophysical, brown: transport, blue: glycolytic, green: growth). Measured entities are indicated by a green background. Perturbations to the model (as stress [NaCl] or different mutations [I: *hog1Δ*, II: *pfk26/27Δ*, III: *HOG1-att*, IV: *gpd1Δ*, V: *FPS1-Δ1* ]).
(PDF)

Figure S11 Changes in cell density in a batch culture experiment. Cells covered by an ODE model highlighted in yellow.
(PDF)

Figure S12 Agreement between main model variables and experimental data. Concentrations of intracellular glycerol (dashed) and phosphorylated Hog1 in different strains (A: WT, B: *pfk26/27Δ*, C: *HOG1-att*, D: *FPS1-Δ1*, E: *gpd1Δ*, F: *hog1Δ*) following hyperosmotic stress of 0.4 M NaCl at *t* = 0.
(PDF)

Figure S13 Experimental data and simulated model variables, stress 0.4 M NaCl added at *t* = 0. A: phosphorylated Hog1, B: GPD1mRNA, C: Gpd1, D: cell volume, E: abundance of open Fps1, F: intracellular trehalose. Concentrations in A and B are scaled as described in text.
(PDF)

Figure S14 Simulation of model variables, stress 0.4 M NaCl added at *t* = 0. A: extracellular glycerol, B: intracellular glycerol, C: extracellular trehalose, D: extracellular glycerol, E: extracellular ethanol, F: extracellular acetate.
(PDF)

Figure S15 Model Simulations and scaled response coefficients for models of different strains. A,D,G: Model variables (solid line: phosphorylated Hog1, dashed line: intracellular glycerol, dotted: abundance of open Fps1) for wild-type, *gpd1Δ* and *hog1Δ*, respectively. B,E,H: scaled response coefficients of osmoshock dependent parameters on intracellular glycerol for wild-type, *gpd1Δ* and *hog1Δ*, respectively. C,F,I: scaled response coefficients of glycolytic parameters on intracellular pyruvate for wild-type, *gpd1Δ* and *hog1Δ*, respectively.
(PDF)

SBML Model S1 Annotated model of osmoadaptation in wild type.
(XML)

SBML Model S2 Annotated model of osmoadaptation in *pfk26/27Δ*.
(XML)

SBML Model S3 Annotated model of osmoadaptation in *HOG1-att*.
(XML)

SBML Model S4 Annotated model of osmoadaptation in *FPS1-Δ1*.
(XML)

SBML Model S5 Annotated model of osmoadaptation in *gpd1Δ*.
(XML)

SBML Model S6 Annotated model of osmoadaptation in *hog1Δ*.
(XML)

Table S1 Optical density (OD) and cell density (CD) for control and stressed WT cultures. OD in arbitrary units, cell density in 10^6^ cells/ml.
(PDF)

Text S1 Materials and Methods, data processing, and details concerning modeling, parameter estimation, and response coefficients.
(PDF)

## References

[1] Yancey PH (2005) Organic osmolytes as compatible, metabolic and counteracting cytoprotectants in high osmolarity and other stresses. J Exp Biol 208: 2819–2830. 16043587 10.1242/jeb.01730

[2] Klipp E, Nordlander B, Kruger R, Gennemark P, Hohmann S (2005) Integrative model of the response of yeast to osmotic shock. Nat Biotechnol 23: 975–982. 16025103 10.1038/nbt1114

[3] Mettetal JT, Muzzey D, Gomez-Uribe C, van Oudenaarden A (2008) The frequency dependence of osmo-adaptation in Saccharomyces cerevisiae. Science 319: 482–484. 18218902 10.1126/science.1151582 PMC2916730

[4] Zi Z, Liebermeister W, Klipp E (2010) A quantitative study of the Hog1 MAPK response to fluctuating osmotic stress in Saccharomyces cerevisiae. PLoS One 5: e9522. 20209100 10.1371/journal.pone.0009522 PMC2831999

[5] Schaber J, Baltanas R, Bush A, Klipp E, Colman-Lerner A (2012) Modelling reveals novel roles of two parallel signalling pathways and homeostatic feedbacks in yeast. Mol Syst Biol 8: 622. 23149687 10.1038/msb.2012.53 PMC3531907

[6] Ansell R, Granath K, Hohmann S, Thevelein JM, Adler L (1997) The two isoenzymes for yeast NAD+-dependent glycerol 3-phosphate dehydrogenase encoded by GPD1 and GPD2 have distinct roles in osmoadaptation and redox regulation. EMBO J 16: 2179–2187. 9171333 10.1093/emboj/16.9.2179 PMC1169820

[7] Luyten K, Albertyn J, Skibbe WF, Prior BA, Ramos J, et al (1995) Fps1, a yeast member of the MIP family of channel proteins, is a facilitator for glycerol uptake and efflux and is inactive under osmotic stress. EMBO J 14: 1360–1371. 7729414 10.1002/j.1460-2075.1995.tb07122.x PMC398221

[8] Tamas MJ, Luyten K, Sutherland FC, Hernandez A, Albertyn J, et al (1999) Fps1p controls the accumulation and release of the compatible solute glycerol in yeast osmoregulation. Mol Microbiol 31: 1087–1104. 10096077 10.1046/j.1365-2958.1999.01248.x

[9] Albertyn J, Hohmann S, Prior BA (1994) Characterization of the osmotic-stress response in Saccharomyces cerevisiae: osmotic stress and glucose repression regulate glycerol-3-phosphate dehydrogenase independently. Curr Genet 25: 12–18. 8082159 10.1007/BF00712960

[10] Påhlman AK, Granath K, Ansell R, Hohmann S, Adler L (2001) The yeast glycerol 3-phosphatases Gpp1p and Gpp2p are required for glycerol biosynthesis and differentially involved in the cellular responses to osmotic, anaerobic, and oxidative stress. J Biol Chem 276: 3555–3563. 11058591 10.1074/jbc.M007164200

[11] Valadi A, Granath K, Gustafsson L, Adler L (2004) Distinct intracellular localization of Gpd1p and Gpd2p, the two yeast isoforms of NAD+-dependent glycerol-3-phosphate dehydrogenase, explains their different contributions to redox-driven glycerol production. J Biol Chem 279: 39677–39685. 15210723 10.1074/jbc.M403310200

[12] Albertyn J, Hohmann S, Thevelein JM, Prior BA (1994) GPD1, which encodes glycerol-3-phosphate dehydrogenase, is essential for growth under osmotic stress in Saccharomyces cerevisiae, and its expression is regulated by the high-osmolarity glycerol response pathway. Mol Cell Biol 14: 4135–4144. 8196651 10.1128/mcb.14.6.4135-4144.1994 PMC358779

[13] Rep M, Albertyn J, Thevelein JM, Prior BA, Hohmann S (1999) Different signalling pathways contribute to the control of GPD1 gene expression by osmotic stress in Saccharomyces cerevisiae. Microbiology 145 Pt 3: 715–727. 10217506 10.1099/13500872-145-3-715

[14] Norbeck J, Påhlman AK, Akhtar N, Blomberg A, Adler L (1996) Purification and characterization of two isoenzymes of DL-glycerol-3-phosphatase from Saccharomyces cerevisiae. Identification of the corresponding GPP1 and GPP2 genes and evidence for osmotic regulation of Gpp2p expression by the osmosensing mitogen-activated protein kinase signal transduction pathway. J Biol Chem 271: 13875–13881. 8662716 10.1074/jbc.271.23.13875

[15] Hirayama T, Maeda T, Saito H, Shinozaki K (1995) Cloning and characterization of seven cDNAs for hyperosmolarity-responsive (HOR) genes of Saccharomyces cerevisiae. Mol Gen Genet 249: 127–138. 7500933 10.1007/BF00290358

[16] Remize F, Barnavon L, Dequin S (2001) Glycerol export and glycerol-3-phosphate dehydrogenase, but not glycerol phosphatase, are rate limiting for glycerol production in Saccharomyces cerevisiae. Metab Eng 3: 301–312. 11676566 10.1006/mben.2001.0197

[17] Dihazi H, Kessler R, Eschrich K (2004) High osmolarity glycerol (HOG) pathway-induced phosphorylation and activation of 6-phosphofructo-2-kinase are essential for glycerol accumulation and yeast cell proliferation under hyperosmotic stress. J Biol Chem 279: 23961–23968. 15037628 10.1074/jbc.M312974200

[18] Tamas MJ, Karlgren S, Bill RM, Hedfalk K, Allegri L, et al (2003) A short regulatory domain restricts glycerol transport through yeast Fps1p. J Biol Chem 278: 6337–6345. 12486125 10.1074/jbc.M209792200

[19] Ferreira C, van Voorst F, Martins A, Neves L, Oliveira R, et al (2005) A member of the sugar transporter family, Stl1p is the glycerol/H+ symporter in Saccharomyces cerevisiae. Mol Biol Cell 16: 2068–2076. 15703210 10.1091/mbc.E04-10-0884 PMC1073684

[20] Rep M, Krantz M, Thevelein JM, Hohmann S (2000) The transcriptional response of Saccharomyces cerevisiae to osmotic shock. Hot1p and Msn2p/Msn4p are required for the induction of subsets of high osmolarity glycerol pathway-dependent genes. J Biol Chem 275: 8290–8300. 10722658 10.1074/jbc.275.12.8290

[21] Westfall PJ, Patterson JC, Chen RE, Thorner J (2008) Stress resistance and signal fidelity independent of nuclear MAPK function. Proc Natl Acad Sci U S A 105: 12212–12217. 18719124 10.1073/pnas.0805797105 PMC2518827

[22] Kuhn C, Petelenz E, Nordlander B, Schaber J, Hohmann S, et al (2008) Exploring the impact of osmoadaptation on glycolysis using time-varying response-coefficients. Genome Inform 20: 77–90. 19425124

[23] Schaber J, Klipp E (2008) Short-term volume and turgor regulation in yeast. Essays Biochem 45: 147–159 doi: 10.1042/BSE0450147 18793130 10.1042/BSE0450147

[24] Schaber J, Adrover MA, Eriksson E, Pelet S, Petelenz-Kurdziel E, et al (2010) Biophysical properties of Saccharomyces cerevisiae and their relationship with HOG pathway activation. Eur Biophys J 39: 1547–1556. 20563574 10.1007/s00249-010-0612-0 PMC2943578

[25] Teusink B, Passarge J, Reijenga CA, Esgalhado E, van der Weijden CC, et al (2000) Can yeast glycolysis be understood in terms of in vitro kinetics of the constituent enzymes? Testing biochemistry. Eur J Biochem 267: 5313–5329. 10951190 10.1046/j.1432-1327.2000.01527.x

[26] Clotet J, Escote X, Adrover MA, Yaakov G, Gari E, et al (2006) Phosphorylation of Hsl1 by Hog1 leads to a G2 arrest essential for cell survival at high osmolarity. Embo J 25: 2338–2346. 16688223 10.1038/sj.emboj.7601095 PMC1478172

[27] Escote X, Zapater M, Clotet J, Posas F (2004) Hog1 mediates cell-cycle arrest in G1 phase by the dual targeting of Sic1. Nat Cell Biol 6: 997–1002. 15448699 10.1038/ncb1174

[28] Gasch AP, Spellman PT, Kao CM, Carmel-Harel O, Eisen MB, et al (2000) Genomic expression programs in the response of yeast cells to environmental changes. Mol Biol Cell 11: 4241–4257. 11102521 10.1091/mbc.11.12.4241 PMC15070

[29] Causton HC, Ren B, Koh SS, Harbison CT, Kanin E, et al (2001) Remodeling of yeast genome expression in response to environmental changes. Mol Biol Cell 12: 323–337. 11179418 10.1091/mbc.12.2.323 PMC30946

[30] Nordlander B, Krantz M, Hohmann S (2008) Hog1-mediated Metabolic Adjustments Following Hyperosmotic Shock in the Yeast Saccharomyces cerevisiae. In: Posas F, Nebreda AR, editors. Stress-Activated Protein Kinases. Berlin/Heidelberg: Springer. pp. 141–158.

[31] Rep M, Reiser V, Gartner U, Thevelein JM, Hohmann S, et al (1999) Osmotic stress-induced gene expression in Saccharomyces cerevisiae requires Msn1p and the novel nuclear factor Hot1p. Mol Cell Biol 19: 5474–5485. 10409737 10.1128/mcb.19.8.5474 PMC84389

[32] Ou X, Ji C, Han X, Zhao X, Li X, et al (2006) Crystal structures of human glycerol 3-phosphate dehydrogenase 1 (GPD1). J Mol Biol 357: 858–869. 16460752 10.1016/j.jmb.2005.12.074

[33] Muzzey D, Gomez-Uribe CA, Mettetal JT, van Oudenaarden A (2009) A systems-level analysis of perfect adaptation in yeast osmoregulation. Cell 138: 160–171. 19596242 10.1016/j.cell.2009.04.047 PMC3109981

[34] Ingalls BP, Sauro HM (2003) Sensitivity analysis of stoichiometric networks: an extension of metabolic control analysis to non-steady state trajectories. J Theor Biol 222: 23–36. 12699732 10.1016/s0022-5193(03)00011-0

[35] Kuhn C, Klipp E (2012) Zooming in on yeast osmoadaptation. Adv Exp Med Biol 736: 293–310. 22161336 10.1007/978-1-4419-7210-1_17

[36] Mollapour M, Piper PW (2007) Hog1 Mitogen-Activated Protein Kinase Phosphorylation Targets the Yeast Fps1 Aquaglyceroporin for Endocytosis, Thereby Rendering Cells Resistant to Acetic Acid. Mol Cell Biol 27: 6446–6456. 17620418 10.1128/MCB.02205-06 PMC2099610

[37] Thorsen M, Di Y, Tängemo C, Morillas M, Ahmadpour D, et al (2006) The MAPK Hog1p Modulates Fps1p-dependent Arsenite Uptake and Tolerance in Yeast. Mol Biol Cell 17: 4400–4410. 16885417 10.1091/mbc.E06-04-0315 PMC1635360

[38] Beese SE, Negishi T, Levin DE (2009) Identification of positive regulators of the yeast fps1 glycerol channel. PLoS Genet 5: e1000738. 19956799 10.1371/journal.pgen.1000738 PMC2773846

[39] Davis DJ, Burlak C, Money NP (2000) Osmotic pressure of fungal compatible osmolytes. Mycological Research 104: 800–804.

[40] Pagnotta SE, McLain SE, Soper AK, Bruni F, Ricci MA (2010) Water and trehalose: how much do they interact with each other? J Phys Chem B 114 14: 4904–4908. 20297794 10.1021/jp911940h

[41] Pereira CS, Hunenberger PH (2008) Effect of trehalose on a phospholipid membrane under mechanical stress. Biophys J 95: 3525–3534. 18599628 10.1529/biophysj.108.131656 PMC2553110

[42] Nordlander B, Krantz M, Hohmann S (2008) Hog1-mediated Metabolic Adjustments Following Hyperosmotic Shock in the Yeast Saccharomyces cerevisiae. Stress-Activated Protein Kinases. Berlin/Heidelberg: Springer pp. 141–158.

[43] Bruggeman FJ, de Haan J, Hardin H, Bouwman J, Rossell S, et al (2006) Time-dependent hierarchical regulation analysis: deciphering cellular adaptation. Syst Biol (Stevenage) 153: 318–322. 16986307 10.1049/ip-syb:20060027

[44] Duch A, de Nadal E, Posas F (2012) The p38 and Hog1 SAPKs control cell cycle progression in response to environmental stresses. FEBS Lett 586: 2925–2931. 22820251 10.1016/j.febslet.2012.07.034

[45] Thomas BJ, Rothstein RJ (1989) Elevated recombination rates in transcriptionally active DNA. Cell 56: 619–630. 2645056 10.1016/0092-8674(89)90584-9

[46] Maiwald T, Timmer J (2008) Dynamical modeling and multi-experiment fitting with PottersWheel. Bioinformatics 24: 2037–2043. 18614583 10.1093/bioinformatics/btn350 PMC2530888

[47] Zi Z, Klipp E (2006) SBML-PET: a Systems Biology Markup Language-based parameter estimation tool. Bioinformatics 22: 2704–2705. 16926221 10.1093/bioinformatics/btl443

