# Correction: Quantitative Analysis of Glycerol Accumulation, Glycolysis and Growth under Hyper Osmotic Stress

**DOI:** 10.1371/journal.pcbi.1003663

**Published:** 2014-05-22

**Authors:** The PLOS Computational Biology Staff

The legends for Supporting Information SBML Models S3, S4 and S6 do not correspond to the correct files. The corrected legends and associated files can be found below.

Inside each SBML file, the Model ID and name attributes give a short description of the cell type described.

## Supporting Information

SBML Model S3 Annotated model of osmoadaptation in *hog1Δ*.
(XML)

SBML Model S4 Annotated model of osmoadaptation in *HOG1-att*.
(XML)

SBML Model S6 Annotated model of osmoadaptation in *FPS1-Δ1*.
(XML)
