# Supplementary figures and images for: Quantitative Analysis of Glycerol Accumulation, Glycolysis and Growth under Hyper Osmotic Stress
Source: PLoS Comput Biol. 2013 Jun 6;9(6):e1003084. doi: 10.1371/journal.pcbi.1003084 (PMC3677637; doi:10.1371/journal.pcbi.1003084)

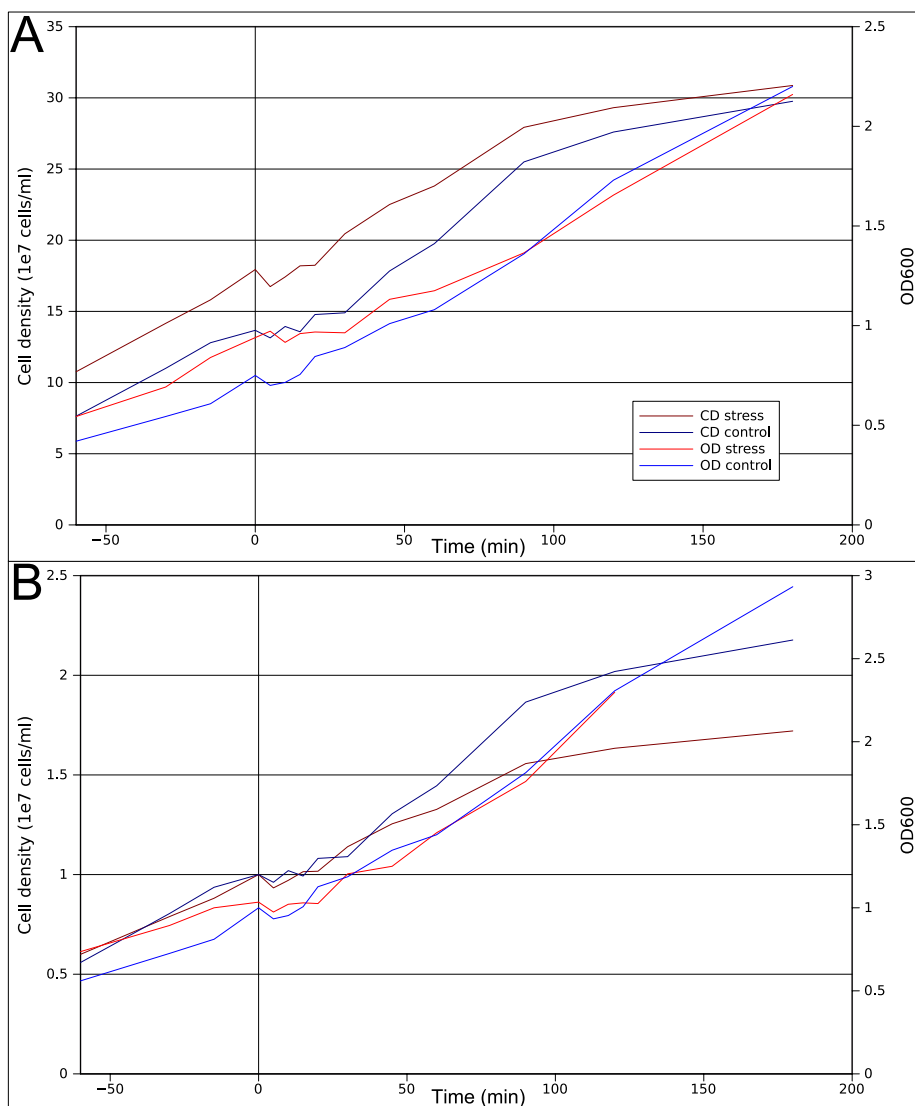

Supplement: Figure S1 — Cell density and optical density in stressed (0.4 M NaCl added at t = 0) and control experiments (A) and normalized to the values at t = 0 (B). Experimental setup as explained in main text. (PDF) [file pcbi.1003084.s008.pdf]

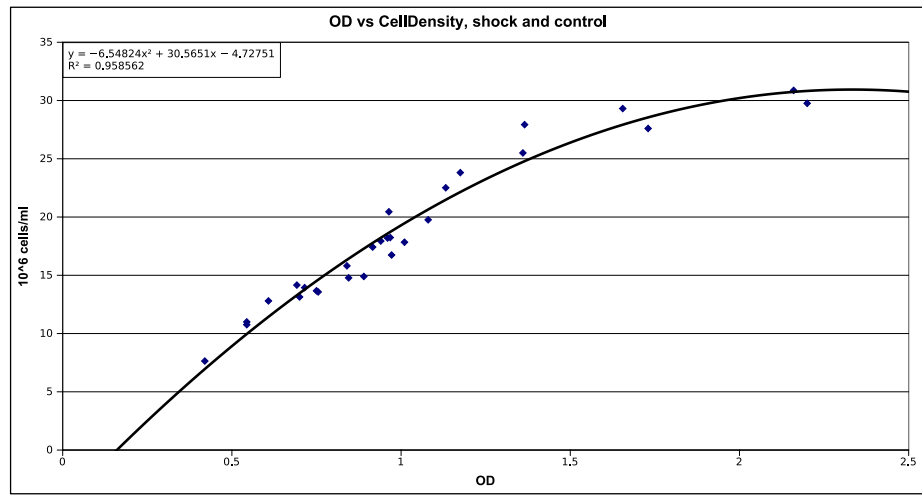

Supplement: Figure S2 — Plot of OD versus cell density values as obtained from control and stressed time course experiments and the function for computing cell density from OD. (PDF) [file pcbi.1003084.s009.pdf]

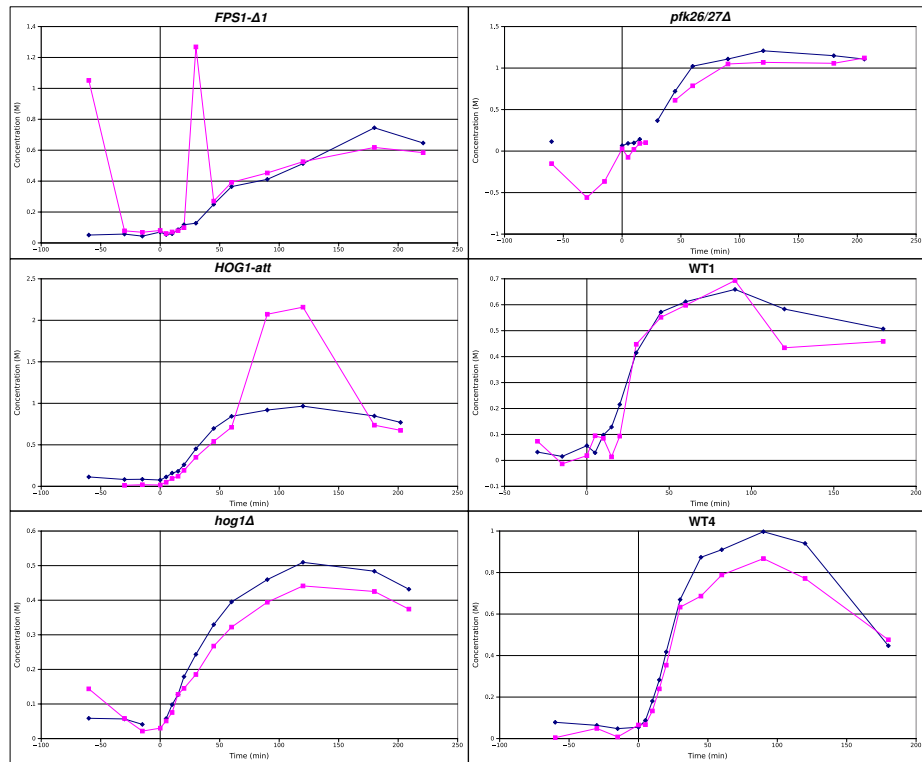

Supplement: Figure S3 — Measured (blue) and inferred (pink) intracellular glycerol for different experiments. Errors in the inferred values due to inconsistencies in measurements are visible in FPS1-Δ1, hog1Δ and WT1. (PDF) [file pcbi.1003084.s010.pdf]

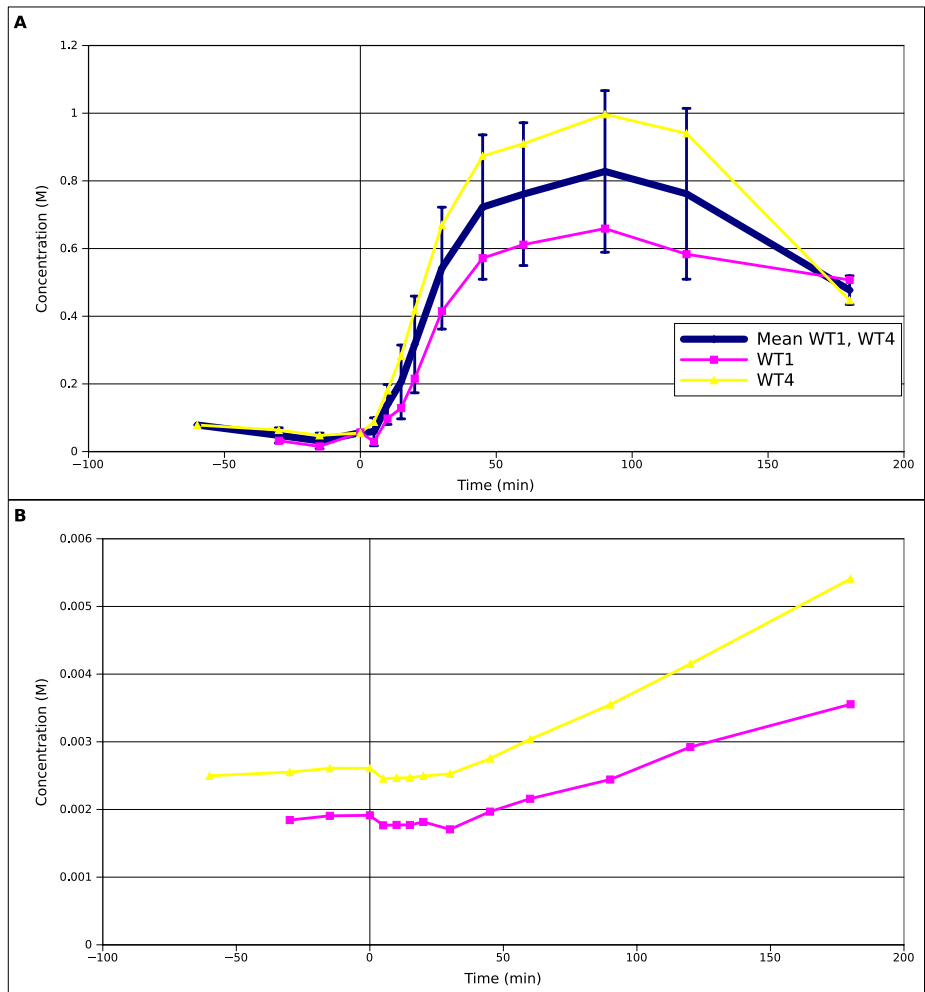

Supplement: Figure S4 — Comparison of glycerol time courses for WT1 and WT4. (A) intracellular glycerol, (B) extracellular glycerol. (PDF) [file pcbi.1003084.s011.pdf]

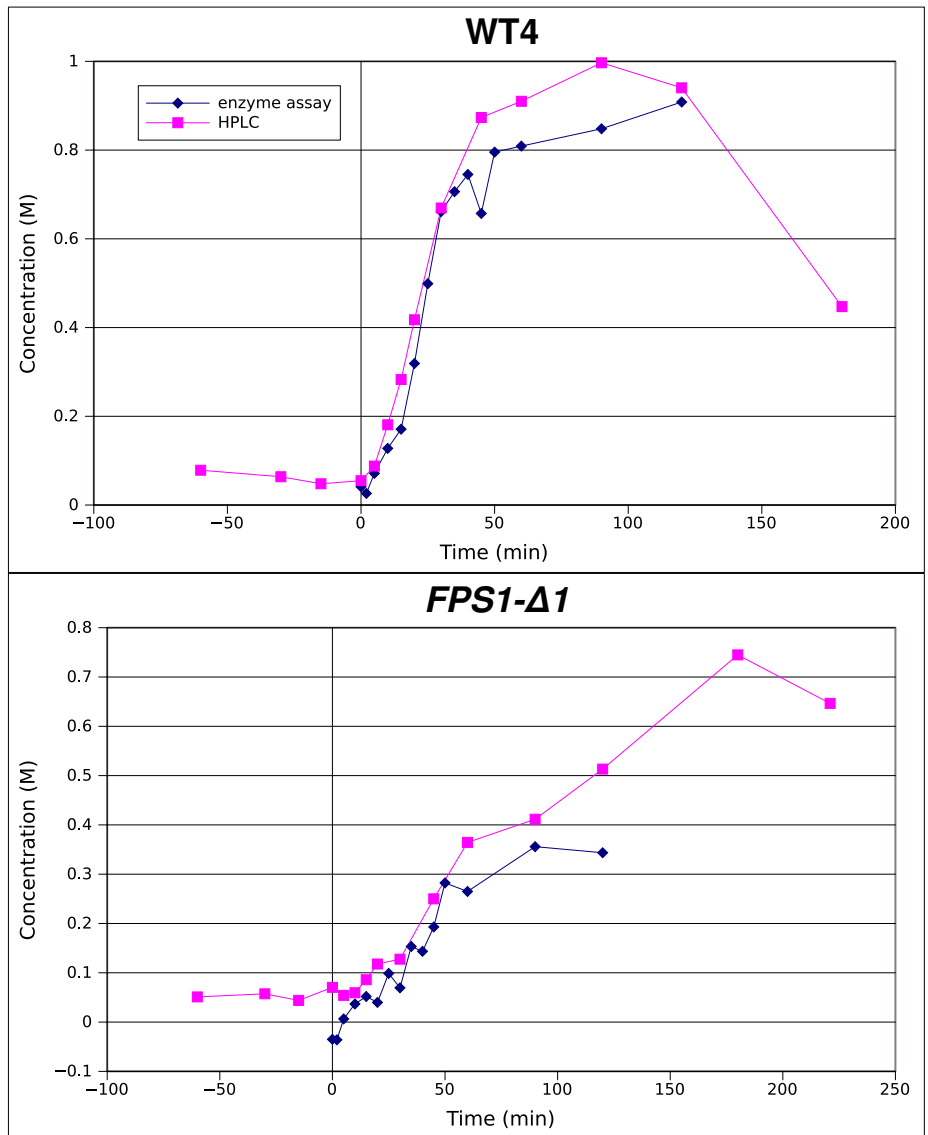

Supplement: Figure S5 — Comparison of enzyme assay (blue) and HPLC (pink) intracellular glycerol quantifications. Underlying data is given in Supplemental Dataset S2. (PDF) [file pcbi.1003084.s012.pdf]

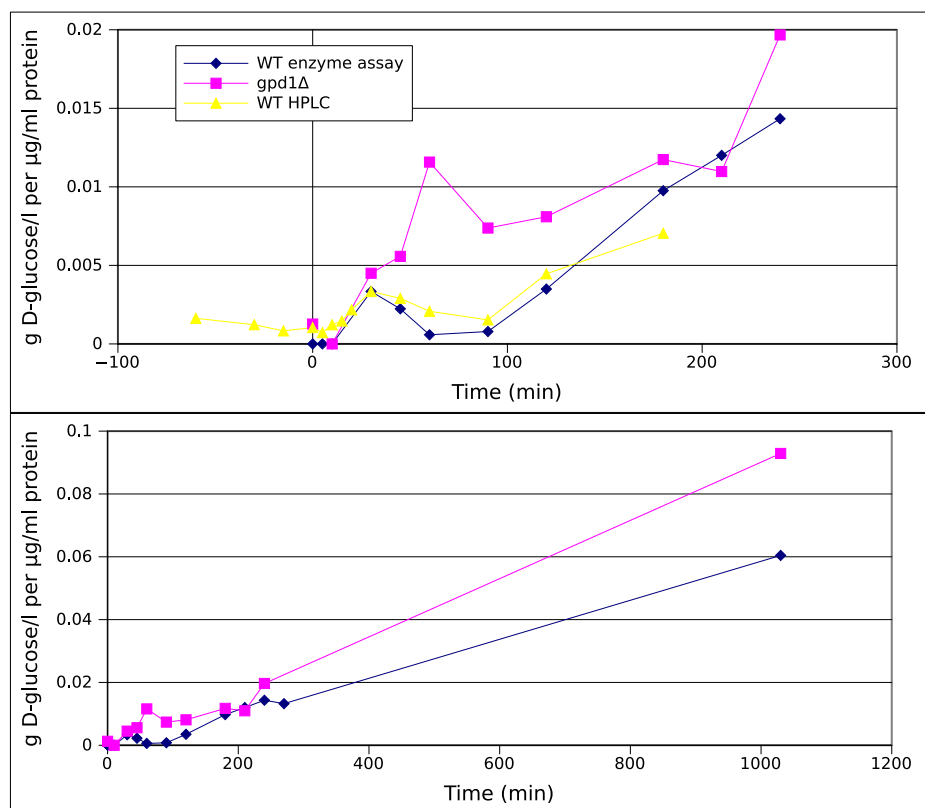

Supplement: Figure S6 — Comparison of intracellular trehalose levels obtained with different methods. Enzyme assay data is in g D-glucose/l per µg protein/ml, HPLC data in mol/l is normalized to the enzyme assay value at t = 30. Underlying data is given in Supplemental Dataset S3. (PDF) [file pcbi.1003084.s013.pdf]

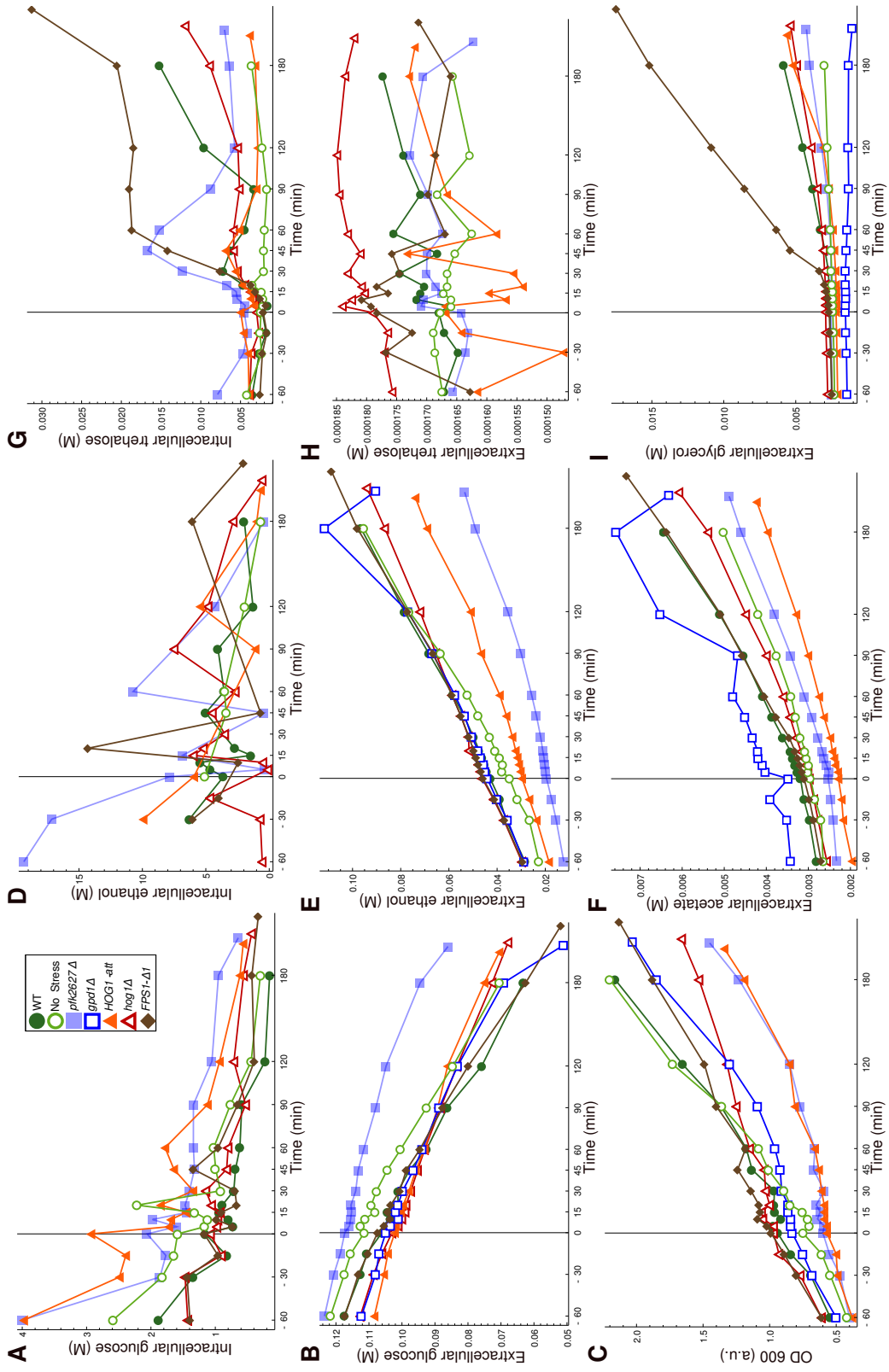

Supplement: Figure S7 — Experimental data as used for model fitting. Measured entities are indicated on y-axes. Shown are representative experiments for each strain. Stress of 0.4 M NaCl is added at t = 0. A: intracellular glucose, B: extracellular glucose, C: Optical density, D: intracellular ethanol, E: extracellular ethanol, F: extracellular acetate, G: intracellular trehalose, H: extracellular trehalose, I: extracellular glycerol. (PDF) [file pcbi.1003084.s014.pdf]

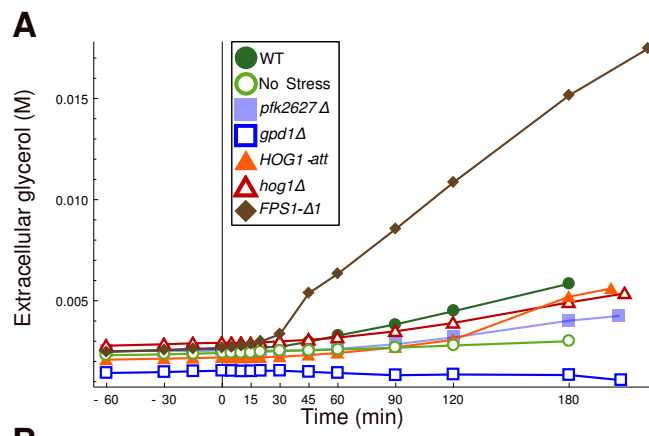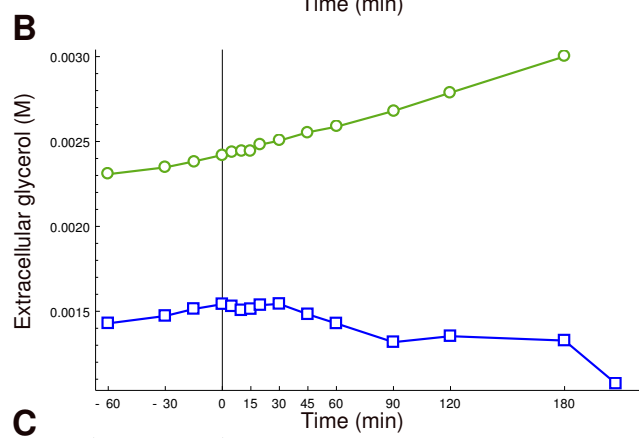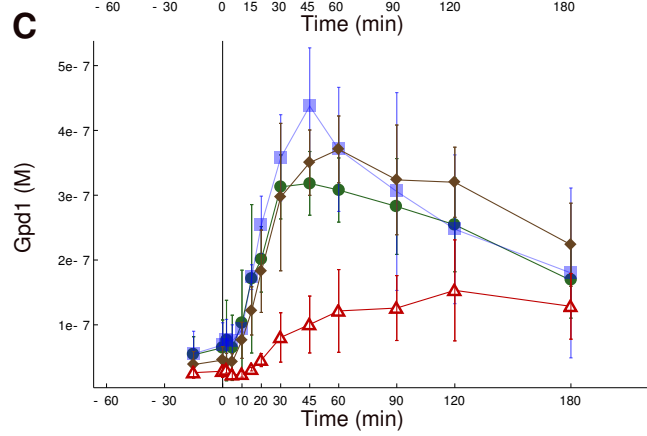

Supplement: Figure S8 — Experimental data for extracellular glycerol (A,B) and Gpd1 (C) following addition of 0.4 M NaCl at t = 0. Close inspection reveals a decrease of extracellular glycerol in gpd1Δ strain. The decrease intracellular glycerol in gpd1Δ is on a similar timescale as the transcriptionally regulated increase of Gpd1 in other strains, indicating that a transcriptionally regulated mechanism is responsible for this decrease as well. (PDF) [file pcbi.1003084.s015.pdf]

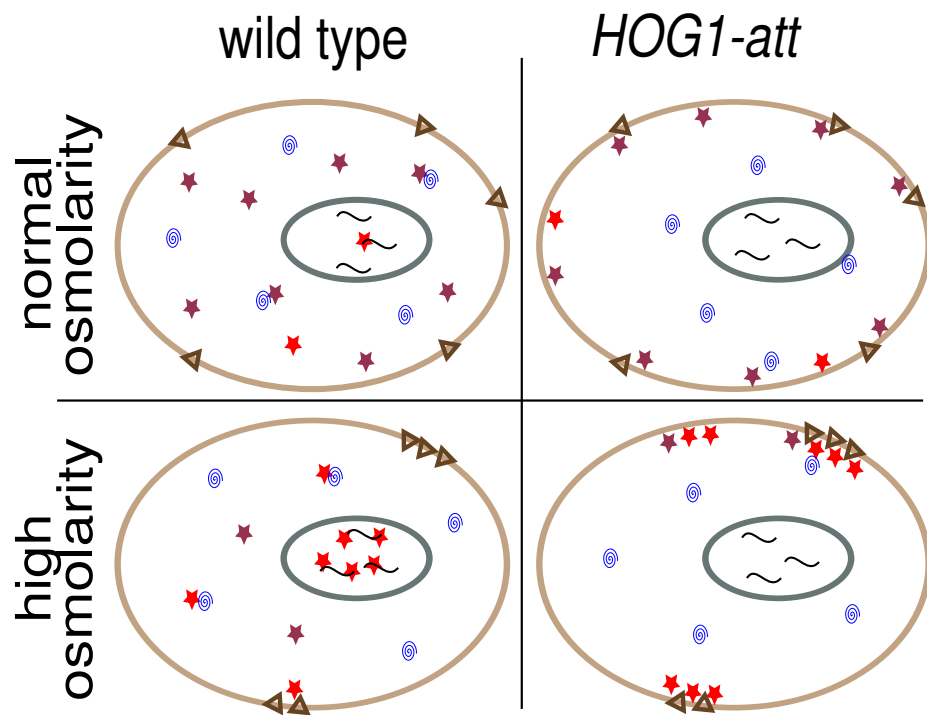

Supplement: Figure S9 — Effects of HOG1-att (Hog1 attached to the plasma membrane) compared to wild-type. Under unstressed conditions, inactive Hog1 (dark red stars) is localized throughout the cell, residual active Hog1 (light red stars) is localized in the nucleus. Possible Hog1 interaction partners are depicted: Fps1 (brown triangles), Pfk26/27 (blue spirals) and genes (black waves). In wild-type, osmoadaptation leads to active Hog1 translocating to the nucleus to stimulate transcription. In HOG1-att, transcriptional regulation is abolished and cytosolic Hog1-concentration is reduced while possible interactions with membrane-bound proteins are increased. (PDF) [file pcbi.1003084.s016.pdf]

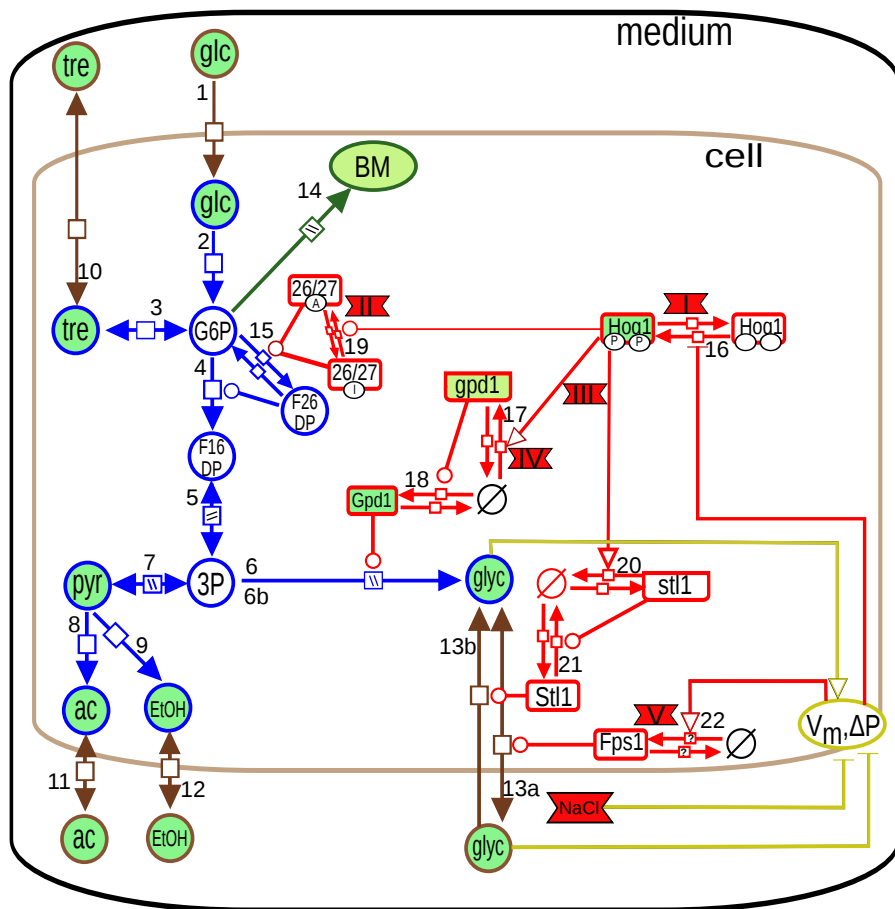

Supplement: Figure S10 — Model topology in SBGN syntax. The different modules are color coded (red: adaptation, yellow: biophysical, brown: transport, blue: glycolytic, green: growth). Measured entities are indicated by a green background. Perturbations to the model (as stress [NaCl] or different mutations [I: hog1Δ, II: pfk26/27Δ, III: HOG1-att, IV: gpd1Δ, V: FPS1-Δ1 ]). (PDF) [file pcbi.1003084.s017.pdf]

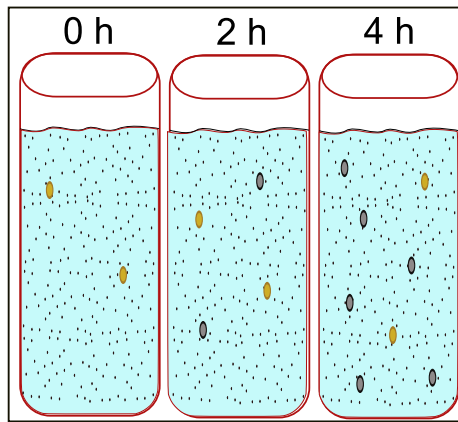

Supplement: Figure S11 — Changes in cell density in a batch culture experiment. Cells covered by an ODE model highlighted in yellow. (PDF) [file pcbi.1003084.s018.pdf]

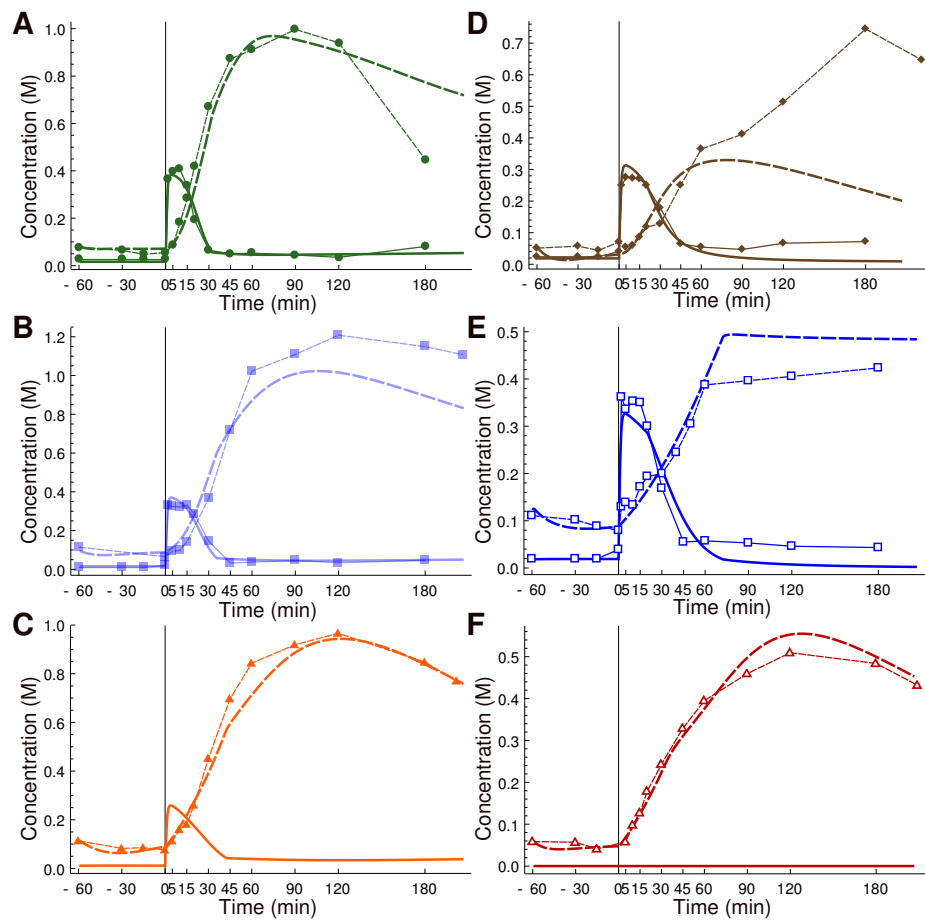

Supplement: Figure S12 — Agreement between main model variables and experimental data. Concentrations of intracellular glycerol (dashed) and phosphorylated Hog1 in different strains (A: WT, B: pfk26/27Δ, C: HOG1-att, D: FPS1-Δ1, E: gpd1Δ, F: hog1Δ) following hyperosmotic stress of 0.4 M NaCl at t = 0. (PDF) [file pcbi.1003084.s019.pdf]

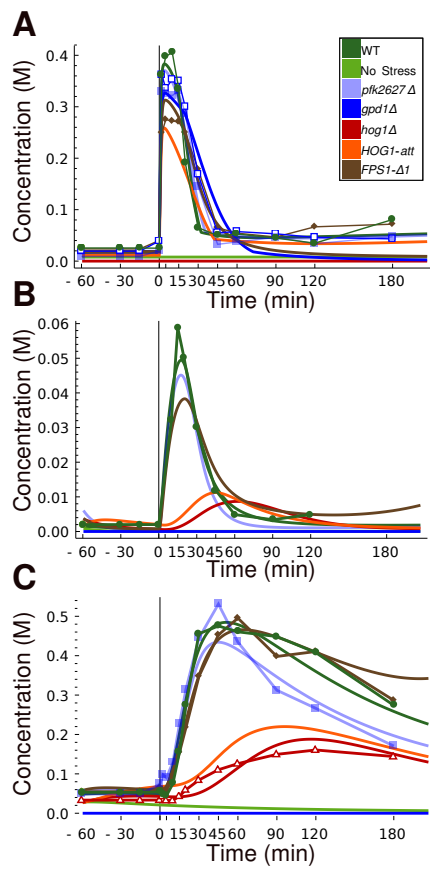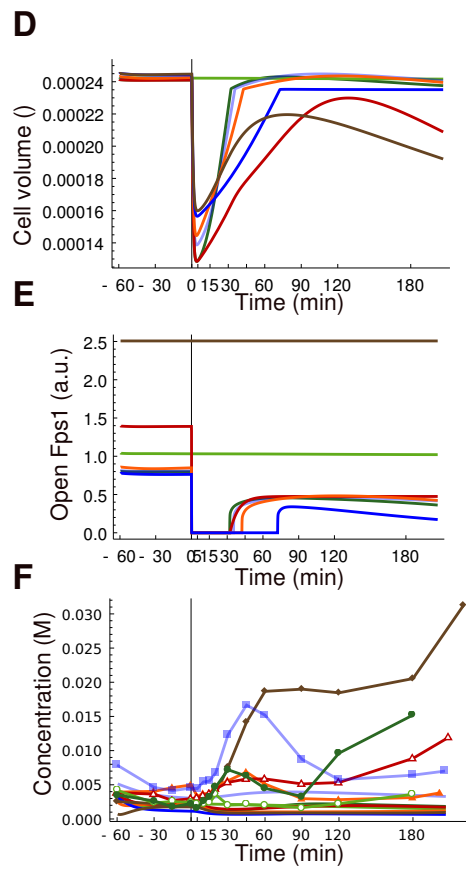

Supplement: Figure S13 — Experimental data and simulated model variables, stress 0.4 M NaCl added at t = 0. A: phosphorylated Hog1, B: GPD1mRNA, C: Gpd1, D: cell volume, E: abundance of open Fps1, F: intracellular trehalose. Concentrations in A and B are scaled as described in text. (PDF) [file pcbi.1003084.s020.pdf]

**A**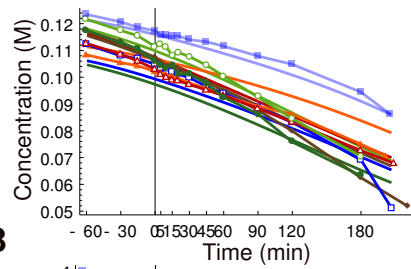**B**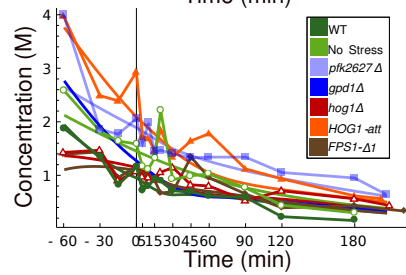**C**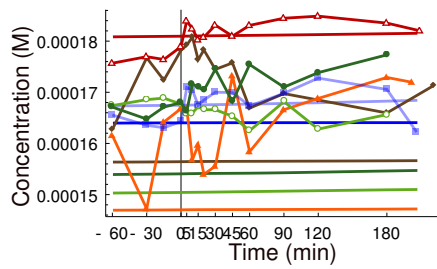**D**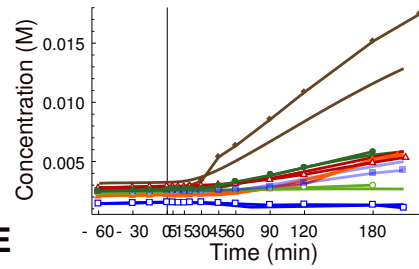**E**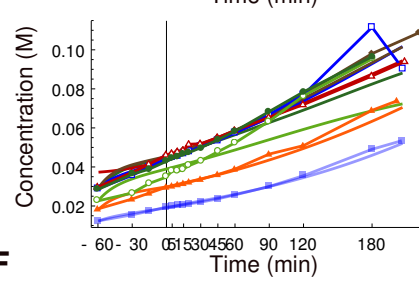**F**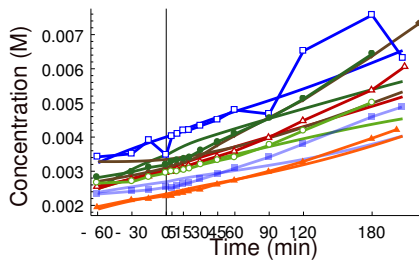

Supplement: Figure S14 — Simulation of model variables, stress 0.4 M NaCl added at t = 0. A: extracellular glycerol, B: intracellular glycerol, C: extracellular trehalose, D: extracellular glycerol, E: extracellular ethanol, F: extracellular acetate. (PDF) [file pcbi.1003084.s021.pdf]

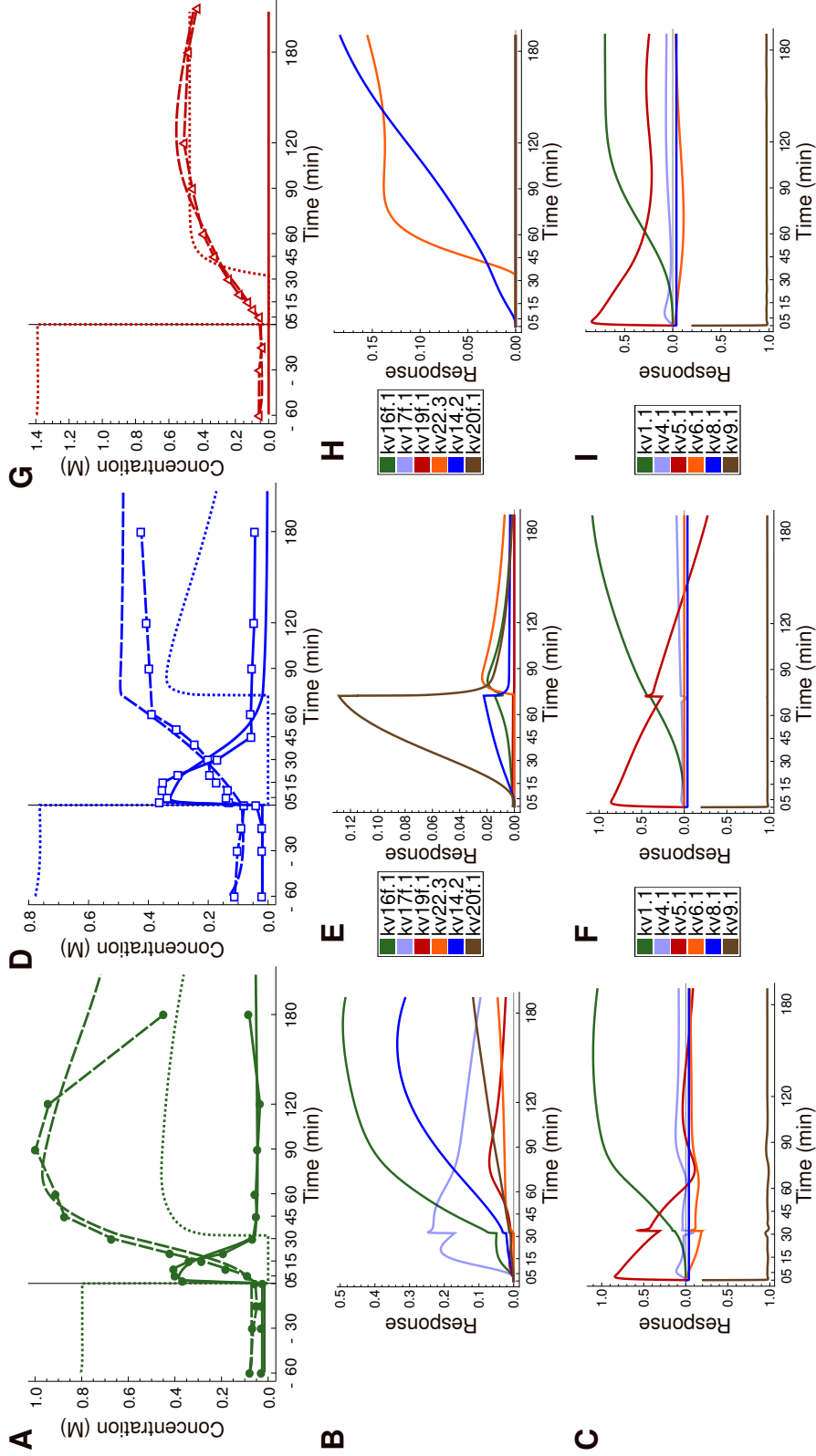

Supplement: Figure S15 — Model Simulations and scaled response coefficients for models of different strains. A,D,G: Model variables (solid line: phosphorylated Hog1, dashed line: intracellular glycerol, dotted: abundance of open Fps1) for wild-type, gpd1Δ and hog1Δ, respectively. B,E,H: scaled response coefficients of osmoshock dependent parameters on intracellular glycerol for wild-type, gpd1Δ and hog1Δ, respectively. C,F,I: scaled response coefficients of glycolytic parameters on intracellular pyruvate for wild-type, gpd1Δ and hog1Δ, respectively. (PDF) [file pcbi.1003084.s022.pdf]
