# Supplementary material for: Quantitative Analysis of Glycerol Accumulation, Glycolysis and Growth under Hyper Osmotic Stress
Source: PLoS Comput Biol. 2013 Jun 6;9(6):e1003084. doi: 10.1371/journal.pcbi.1003084 (PMC3677637; doi:10.1371/journal.pcbi.1003084)
Supplement: Table S1 — Optical density (OD) and cell density (CD) for control and stressed WT cultures. OD in arbitrary units, cell density in 106 cells/ml. (PDF) [file pcbi.1003084.s029.pdf]

	Control		0.4M NaCl	
Time (min)	OD	CD	OD	CD
-60	0.42	7.64	0.544	10.76
-30	0.544	11	0.692	14.16
-15	0.608	12.8	0.84	15.81
0	0.75	13.67	0.94	17.94
5	0.7	13.14	0.972	16.74
10	0.715	13.94	0.916	17.42
15	0.755	13.57	0.96	18.2
20	0.845	14.78	0.968	18.24
30	0.89	14.9	0.964	20.45
45	1.01	17.84	1.132	22.51
60	1.08	19.76	1.175	23.81
90	1.36	25.5	1.365	27.93
120	1.73	27.6	1.655	29.31
180	2.2	29.76	2.16	30.87
