# Supplementary material for: Quantitative Analysis of Glycerol Accumulation, Glycolysis and Growth under Hyper Osmotic Stress
Source: PLoS Comput Biol. 2013 Jun 6;9(6):e1003084. doi: 10.1371/journal.pcbi.1003084 (PMC3677637; doi:10.1371/journal.pcbi.1003084)
Supplement: Text S1 — Materials and Methods, data processing, and details concerning modeling, parameter estimation, and response coefficients. (PDF) [file pcbi.1003084.s030.pdf]

Text S1
Quantitative analysis of glycerol accumulation, glycolysis and growth under hyperosmotic stress

Elzbieta Petelenz-Kurdziel, Clemens Kühn,
Bodil Nordlander, Dagmara Klein, Kuk-Ki Hong,
Therese Jacobson, Peter Dahl, Jörg Schaber,
Jens Nielsen, Stefan Hohmann, Edda Klipp

Contents

1 Material and Methods **2**

2 Experimental Data and Data Processing **3**

3 Modeling Details **5**

4 Parameter Estimation Details **13**

5 Modifications to improve model of *FPS1*-Δ*1* **13**

6 Response Coefficients **14**

1 Material and Methods

primary antibody – yC20 total Hog1 polyclonal goat antibody (Santa Cruz Biotechnology Inc.), 1:2 000 in Odyssey Blocking Buffer with TBST (1:1000), 1 h at room temperature, third primary antibody – rabbit polyclonal antisera Gpd1-A (Innovagen), 1:2 000 in Odyssey Blocking Buffer with TBST (1:1 000), 1 h at room temperature, and simultaneously with secondary antibodies: donkey anti-goat IR Dye 680, 1:12 500 and donkey anti-rabbit IR Dye 800CW 1:12 500 (Li-Cor Biosciences), in Odyssey Blocking Buffer with TBST (1:1000), for 45 min at room temperature. The membranes were scanned using Odyssey Infrared Imaging System (Li-Cor Biosciences) and quantified using Multi Gauge 3.0 (FujiFilm) software. Refractometer Millipore, CA, USA) and acetate, succinate and pyruvate by ultraviolet-1 visible light absorbance detector (Waters 486 Tunable Absorbance Detector set at 210 nm, Millipore, CA, USA).

Glycerol Assays

Yeast cells were cultured in YPD medium (Yeast Peptone D-glucose; 1% yeast extract (Bacto), 2% peptone (Bacto), 2% glucose). The cells were grown to exponential phase (OD600=0.7), harvested by centrifugation (13 000 rpm), frozen in liquid nitrogen and stored in -20°C. The frozen pellets were boiled for 10 min with 1 ml of ice-cold water (12 samples at a time) and spun down (1 min at 13 000 rpm). The supernatant was transferred to new tubes and stored in -20°C before further processing. The glycerol content of the samples was analyzed using an enzymatic kit (Roche, Cat. No. 10 148 270 035). 200μl of sample per well were loaded onto a 96 well plate, along with a dilution series of a glycerol content standard (solution 4). Samples were mixed with reagents using a Biomek 2000 robot (Beckman), transferred to another plate containing reagent 2 (2g of coenzyme/buffer mixture [glycylglycine buffer, pH approx. 7.4; NADH, approx. 7 mg; ATP, approx. 22 mg; PEP-CHA, approx. 11 mg; magnesium sulfate] diluted in 11ml redist. water) from the glycerol kit and measured using a plate reader; then the reaction was stopped with reagent 3 (0.4 ml consisting of: pyruvate kinase, approx. 240 U; L-lactate dehydrogenase, approx. 220 U) and the samples were measured again. The glycerol concentrations were determined using the dilution series from the enzymatic kit as a calibration curve. Absorbance was measured at 340 nm.

Trehalose Assays

Trehalose assays were conducted as described in Parrou and Francois, Analytical Biochemistry 1997: cells (4-10 mg dry wt) were collected by centrifugation (3 min at 5000g; 0-4°C), carefully drained to remove the culture medium re-suspended in 0.25 ml of 0.25M Na2CO3 using screw-top Eppendorf tubes and incubated at 95°C for 4h. The mixture was brought to pH 5.2 by addition of 0.15 ml of 1M acetic acid and 0.6 ml of 0.2 M Na-acetate, pH 5.2. Half of the suspension was incubated overnight with trehalase (0.05 U/ml) (Sigma Cat. No. T-8778) at 37°C under constant agitation. The suspensions were centrifuged for 3 min at 5000g. The glucose content was determined using 20 μl (adequately diluted in water) of supernatant by addition of 200 μl of glucose oxidase mixture (Sigma, Cat. No. 510-A) and read at 420 nm in a ELISA reader apparatus. Units: μg glucose equivalents/107 cells

Northern Blots

Yeast cells were cultured in YPD medium (Yeast Peptone D-glucose; 1% yeast extract (Bacto), 2% peptone (Bacto), 2% glucose). Cultures of 500 ml were grown to mid-exponential phase (OD600=0.7). Samples of 10ml were taken at different time points, cooled rapidly by mixing with 40ml of ice-cold MiliQ water and centrifuged for 5 min at 3000 rpm. Pellets were re-suspended in 1ml ice-cold water (MiliQ), transferred into 2ml tubes with screw caps and re-sedimented (13 200 rpm for 20 s). At this stage pellets could be frozen and stored at -20°C. The cell content was extracted from the frozen pellets by bead beating: 500 μ 0.45 mm glass beads, 500 μl extraction buffer, 500 μl PCI (citrate-buffered water-equilibrated phenol pH 4.2, chloroform, isoamyl alcohol, 25:24:1) and 50 μl 10% SDS was added to the pellets. The samples were mixed for 20 s at 6 m/s using a fast prep desiccator Bio101. The extracted samples were centrifuged for 10 min at 14 000 rpm, in 4°C; the aqueous supernatant was transferred into new tubes and extracted with 1 ml 100% ethanol. After at least 1 h cooling in -20°C RNA pellets were obtained by centrifugation (14 000 rpm, 10 min, 4°C). Dried pellets were dissolved overnight in 50 μl of RNase-free water. The quality of the RNA samples was examined using both (i) 1% agarose gel containing formaldehyde and (ii) spectrophotometer.

RNA samples were diluted to 2000 μg/ml, separated on a 1% agarose gel and transferred by capillary blotting into a Hybond N membrane (Amersham) in 10x saline-sodium citrate (SSC) buffer overnight. Subsequently the membrane was rinsed in 2x SSC, cross-linked in a UV cross-linker and incubated with standard hybridization buffer. The probe activity was determined by scintillation, using Nick columns. The membrane was pre-hybridized for at least 2 hours in 68°C, hybridized with a calculated amount (based on the

scintillation measurement) of denatured DNA probe (3 min at 100°C) at 68°C overnight washed in 0.5x SSC with 0.1% SDS (45min in 68°C) and pre-developed for 3 hours; the result was view on the Phosphor Imager using Quantity One software. The actual exposure was done overnight. The membrane was stripped with 0.1% SDS in boiling water (2 rounds 20 min each) and hybridized with another DNA probe.

Additional Western Blots

Yeast cells were cultured in YPD medium (Yeast Peptone D-glucose; 1% yeast extract (Bacto), 2% peptone (Bacto), 2% glucose). Cultures of 500 ml were grown to mid-exponential phase (OD600=0.7). Samples of 4.5 ml were collected at different time points, distributed evenly into three 1.5 ml tubes per each time point and frozen immediately in a dry ice ethanol bath. Fully frozen samples were thawed on ice; cell pellets were harvested by centrifugation and stored at -20°C for further processing. The pellets (three per time point) were pooled using 0.5 ml ice cold water, sedimented by centrifugation, suspended in 40 μl loading buffer (100mM Tris-HCl pH 6.8, 20% glycerol, 200mM DTT, 4% SDS, 10mM NaF, 0.1 mM Na3V04 (sodium orthovanadate), protease inhibitor (Complete EDTA-free Protease Inhibitor Cocktail tablets, Roche), and 20 mM mercapto-ethanol) and purified by centrifugation. Purified protein extracts were stored at -20°C for further processing. The protein content was determined by precipitation with DOC and TCA, using a protein content determination kit (BioRad). Samples containing 20-25 μg proteins with loading buffer (1:1) containing bromophenol blue were electrophoresed on a 10% polyacrylamide gel and transferred over night at 4°C to a nitrocellulose membrane (Hybond-ECL, Amersham) using a BioRad wet transfer system. Membranes were blocked with 5% milk (Difco) in TBST and incubated with antibodies: primary - phospho-p38 MAPK (Thr180/Tyr182) antibody (Cell Signalling), 1:1000 in 5% BSA TBST, over night incubation at 4°C; secondary - anti-rabbit antibody HRP-linked IgG (Cell Signalling), 1:2000 in 5% milk TBST, for 1 h at room temperature. Membranes were developed with Lumi Light Western Blotting Substrate (Roche), scanned using a Fuji Film LAS-1000 CCD camera with Image Reader LAS-1000 Pro V2.6 software and quantified using Multi Gauge 3.0 software.

2 Experimental Data and Data Processing

Metabolite Data

Metabolite levels were quantified over time by HPLC. The time course for wild type cells was performed four times and the control with unstressed wild type cells was performed in duplicate. In addition, we conducted time course experiments for all different strains used in this study (*FPS1-Δ1*, *gpd1*Δ, *HOG1-att*, *hog1*Δ, *pfk26/27*Δ) after adding 0.4 M NaCl.

Cell Density Measurements

Optical density was recorded for each experiment. An additional measurement of cell density was performed for one wild type and one control experiment, respectively. Recorded values are given in Table S1 and Figure S1.

Cell density (in 10^6 cells/ml) was fitted to OD with satisfying precision ($R^2 = 0.959$, see also Figure S2) as

$$CD(OD) = -6.54824 \cdot OD^2 + 20.5651 \cdot OD - 4.72751. \tag{1}$$

This regression allows to calculate the cell density of any given OD measurement with the used spectrophotometer and was used to correct uptake and export rates for the increase in cell density over time.

Data Processing and Inference of Missing Data Points

Raw HPLC results in g/l were processed by dividing by the molar mass to obtain mol/l for extracellular and total samples. For intracellular samples, additional dilution of the medium-free cell fraction in 1 ml water was accounted for: assuming an average cell volume of 50 femtoliter, the intracellular concentration c^{proc}_{intra} is calculated from the measured values c^{raw}_{intra} as

$$c^{proc}_{intra} = \frac{c^{r}_{intra}aw}{molarmass} \cdot \frac{0.001}{CD(t) \cdot 10^6 \cdot 50 \cdot 10^-15} \tag{2}$$

This processing is reproduced in Supplemental Dataset D1.

Samples were collected and measured for total, extra-, and intracellular concentrations. Careful analysis allows the estimation of one of the three concentrations given that the remaining two and the according volumes V are known:

Since the amount (expressed as mass m) of a metabolite in the total sample must equal the sum of the masses in extracellular and intracellular samples, i.e.

$$m_{tot} = m_{in} + m_{ex} \Leftrightarrow c_{tot} \cdot V_{tot} = c_{in} \cdot V_{in} + c_{ex} \cdot V_{ex}, \quad (3)$$

we can calculate the intracellular concentration as

$$c_{in} = \frac{c_{tot} \cdot V_{tot} - c_{ex} \cdot V_{ex}}{V_{in}}. \quad (4)$$

The total volume is 1 ml, the intracellular volume can be extrapolated from OD measurements and is in a range of $\frac{1}{500}$ to $\frac{1}{2000}$ of total volume, so that extracellular volume can be assumed to equal total volume.

This calculation is prone to slight measurement errors, but generally allows inferring missing measurements as shown in Supplemental Dataset D1. Supplemental Figure S3 shows a comparison between estimated and measured glycerol, indicating inconsistencies in estimated data of *FPS1*-Δ*1*, *hog1*Δ and WT1, that could be easily identified.

The high consistency between the measured and inferred data indicates a high reproducibility of our experimental results.

This inference is used to generate a time course for *gpd1*Δ for fitting, for which intracellular glycerol was not measured.

Reproducibility of Wild Type Experiments

The experimental data obtained from different experiments with the wild type strain are in very good agreement, however they differ for internal glycerol between repetitions, i.e. WT1 and WT4 (see Supplemental Figure S3). This difference is due to higher external glycerol in WT 4 (Supplemental Figure S4): The Stl1-mediated uptake of extracellular glycerol and the reduced Fps1-mediated diffusion of glycerol out of the cell lead to the observed differences.

The intracellular glycerol of experiments WT2 and WT3 inferred from extracellular and total measurements are similar to the time course observed for WT1 (compare Supplemental Dataset D1).

These results further support reproducibility of our experimental data and a corresponding decreased glycerol accumulation is observed in model simulations using initial conditions from the WT1 experiment.

Representative Experiments

Each batch culture experiment is affected by slight differences in initial cell density and culture conditions. Since no steady state is reached, these differences can influence the cellular behavior. This is exemplified by the difference between WT4 and WT1 intracellular glycerol levels, as described above. To maintain consistency of datasets used for fitting, all further steps were done using one representative experiment. For wild type, WT4 was chosen because it exhibits the strongest agreement with previous enzyme assay experiments (see Supplemental Figure S5) and cell density measurements had been done in this experiment. Likewise, NoStress2 was chosen as representative experiment for unstressed wild type.

Western Data

Raw Western blot results provided in intensities have been processed as follows:

- Intensities were normalized to the average of the respective wild type experiment to minimize the effect of single measurement errors in normalization.
- Molecules per cell as reported in Ghaemmaghami et al., 2003 have been converted to mol/cell.
- These values are converted to concentrations assuming an intracellular volume of 50 fl.
- For Hog1PP results, we assumed a peak of 90% Hog1PP of total Hog1 in wild type time course, assuming that total Hog1 does not change during osmoadaptation.
- For Gpd1 results, we assumed that the value reported in Ghaemmaghami et al., 2003 as the concentration of Gpd1 before stress.

Details of this processing are given in Supplemental Dataset D2, Charts
Hog1PP_ALL and Gpd1_ALL.

Additional Data

The time course of gpd1mRNA used for fitting (see Supplemental Figure S13) was extracted from Klipp et al., 2005 and scaled. The simulated time course of stl1mRNA qualitatively reproduces experimental quantifications given in Supplemental Dataset D7.

Prior to the HPLC measurements, enzymatic quantifications of glycerol have been carried out. Processing of the raw enzyme assay data according to the processing applied to HPLC measurements indicates a high reproducibility between both data types, as shown in Supplemental Figure S5 for wild type and *FPS1-*Δ*1*.

In addition to the quantification of intracellular trehalose via HPLC, intracellular trehalose levels have been quantified via an enzyme assay as described in Supplemental Material and Methods. This quantification supports the hypothesis that trehalose contributes to long-term adaptation to sustained high osmolarity and is qualitatively similar to the HPLC data, as shown in Supplemental Figure S6.

Additionally, Western blot Hog1PP quantifications, enzymatic glycerol quantifications, enzymatic trehalose quantifications, and Northern blot mRNA quantifications are available in Supplemental Datasets D4, D5, D6 and D7.

Data Compilation

The compiled data as described above has been used to estimate model parameters. An overview of the most important data series is given in Supplemental Figure S7.

For better visualization, the extracellular glycerol concentration in *gpd1*Δ in comparison to control is indicated in Supplemental Figure S8.

HOG1-att

As described in the main text, we infer a regulatory effect of Hog1 on Fps1 from results obtained with the *HOG1-att* strain. In this strain, Hog1 is tethered to the membrane. Direct physical interaction between Hog1 and Fps1 has been reported for different stress conditions (Thorsen et al., 2006 and Mollapour and Piper, 2007). Moreover, the discrepancy between glycerol concentration in model simulations of *hog1*Δ and *HOG1-att* could not be attributed to mechanisms implemented so far. If Hog1 regulates the abundance of open Fps1 under hyperosmotic conditions, mutations in HOG1 do affect the abundance of open Fps1 and its regulation.

In *hog1*Δ strains, an increased basal glycerol efflux compared to wild-type has been observed by Tamas et al., 1999. This is in agreement with our observations and conjectures. Currently, no experimental data is available on the effect of *HOG1-att* on basal glycerol efflux or Fps1 transport rate. However, an apparent effect of *HOG1-att* is an increased concentration of Hog1 at the plasma membrane so that any interactions between membrane-bound effectors and Hog1 are likely increased (compare Supplemental Figure S9).

3 Modeling Details

Here, we present detailed description of the mathematical model. A graphical overview of the model topology in SBGN format is given in Supplemental Figure S10.

Biophysical Changes

Changes in cell volume (basal solid volume, V_b, and an osmotically active volume V_{os}, cell surface area, osmotic pressure (as a function of external osmotic pressure, internal osmolyte concentrations, and turgor pressure) and turgor pressure (as function of V_{os}).

Glycolysis Module

The glycolysis module was constructed on the basis of existing models, e.g. Klipp et al. 2005 and Teusink et al., 2000. In order to reduce the number of parameters in the model and reach a high coverage of model variables by experimental data, glycolysis was simplified compared to more extensive models. While known main regulatory nodes in osmoadaptation were included in the model ($G6P$, $F16DP$, $F26DP$, $triose$) despite lacking data, most other metabolites for which no data was available were removed and the respective reactions were lumped together. This results in mostly unidirectional reactions in this model of glycolysis because each lumped reaction that includes at least one unidirectional reaction is modeled as unidirectional.

Along with unobserved metabolites, we also omitted cofactors such as NADH/NAD and ATP/ADP. This increases model reliability because

- No data on cofactors during hyperosmotic stress is available. Additionally, reliable measurement of these concentrations requires sophisticated methods that are difficult to integrate in this project (see e.g. Canelas et al., 2008 on the determination of cytosolic NADH/NAD concentrations).
- Under steady state conditions, ATP, ADP and NADH, NAD concentrations are constant. In a model describing the temporal dynamics of osmoadaptation, these concentrations can be expected to vary and this would require to model all glycolytic reactions with very high detail as well as modeling all other reactions that consume/produce these cofactors and change during hyperosmotic stress. This list of additional reactions is currently unknown and could comprise membrane and cell wall maintenance reactions as well as energy-dependent ion transport for which no data is available.

Reactions that do not represent major osmo-dependent regulatory steps are described using Michaelis-Menten kinetics: $v_2(t)$, $v_3(t)$, $v_5(t)$, $v_{6b}(t)$, $v_7(t)$, $v_8(t)$, $v_9(t)$ and v_{15r}.

Reaction v_4 ($G6P \rightarrow F16DP$) is described with a Michaelis-Menten kinetics with two different V_{max}: $k_{v4.1}$ and $k_{v4.2}$ for $F26DP$-activated phosphofructokinase and non-activated phosphofructokinase, respectively. Hence, the contribution of either depends on the binding of $F26DP$ to phosphofructokinase such that

$$\begin{aligned} v_4(t) =&(k_{v4.1} \cdot \frac{F26DP(t)^{k_{v4.5}}}{(F26DP(t)+k_{v4.3})^{k_{v4.5}}} \\ &+ k_{v4.2} \cdot (1 - \frac{F26DP(t)^{k_{v4.5}}}{(F26DP(t)+k_{v4.3})^{k_{v4.5}}})) \\ &\cdot \frac{(G6P(t)/k_{v4.4})^8}{1+(G6P(t)/k_{v4.4})^8)} \end{aligned} \tag{5}$$

Reaction v_6 ($triose \rightarrow glycerol$) is described as a Michaelis-Menten kinetics with the enzyme concentration ($Gpd1(t)$) explicitly mentioned.

Transport Module

Glucose transport is described as Michaelis-Menten kinetics. This is a reasonable simplification that reproduces the time course of extracellular glucose for all experiments (see Supplemental Figure S14).

Transport rates of trehalose, acetate and ethanol (v_{10}, v_{11}, v_{12}) are specified as dependent on the gradient of intracellular and extracellular concentrations C_{intra} and C_{extra}:

$$v_{diff}(t) = k_1 \cdot CellSurface(t) \cdot (C_{intra} - k_2 \cdot C_{extra}) \tag{6}$$

where k_1 represents the abundance of transport proteins and k_2 accounts for specific properties of the respective transporter type (where $k_2 = 1$ describes diffusion). Although this description simplifies the underlying biology, it allowed for a concise representation.

Diffusion of glycerol through Fps1 is described accordingly, only taking the abundance of open Fps1 ($Fps1r$) into account. Uptake of glycerol through Stl1 is again described using a Michaelis-Menten kinetics explicitly referencing the concentration of Stl1.

In batch culture experiments, cell density increases, as illustrated in Supplemental Figure S11. Although the cellular state does not change (e.g. intracellular metabolite concentrations), the global transport rates that affect extracellular concentrations are influenced by cell density.

Because ODE models classically describe a fixed number of cells or one single cell, the rates of extracellular concentration changes are multiplied by the ratio of the cell density $a_5(t)$ as computed from OD measurements and the initial cell density, k_{batch}. Because the ratio of extracellular to intracellular volume is about 2000 (see above), the changes of extracellular concentrations are calculated as

$$\pm v_{transport}(t)/2000 \cdot \frac{a_5(t)}{k_{batch}} \tag{7}$$

Biomass and Adaptation Module

The majority of reactions in this module is described using Mass Action kinetics (all translation and degradation reactions, and inactivation of activated compounds): $v_{16r}(t)$, $v_{17r}(t)$, $v_{18f}(t)$, $v_{18r}(t)$, $v_{19r}(t)$, $v_{20r}(t)$, $v_{21f}(t)$, $v_{21r}(t)$, $v_{AOG2r}(t)$.

To simplify the model, the signaling cascades activating Hog1 were not included. This greatly reduces the number of model variables and parameters while Hog1 activation can be faithfully modeled because previous studies show that Hog1 activity is negatively correlated with cell volume (Klipp et al, 2005 and Muzzey et al.,

2009). Accordingly, Hog1 activity is described as Mass Action kinetics dependent on volume: $\left(\frac{k_{v16f.2}}{a_1(t)}\right)^{k_{v16f.3}}$. The exponent $k_{v16f.3}$ is necessary to induce a strong change in Hog1 activity due to a comparably light decrease in cell volume. Since the initial volume is regained in our model but experimentally measured Hog1PP levels do not return to basal levels, we also included a dependency on extracellular osmolarity ($a_2(t)$) such that the rate of Hog1 activation becomes

$$v_{16f}(t) = Hog1(t) \cdot a_2(t) \cdot k_{v16f.1} \cdot \left(\frac{k_{v16f.2}}{a_1(t)}\right)^{k_{v16f.3}}. \tag{8}$$

The rate of activation of the Hog1-independent activator of Gpd1-mediated glycerol production ($AOG2$) is described accordingly, not taking the external osmolarity into account. This activator is necessary to reproduce transcriptional profiles of GPD1 in *hog1*Δ as described in Rep et al., 1999.

GPD1 transcription depends on Hog1PP, the Hog1-independent activator $AOG2$ mentioned above and a basal term. Interaction between Hog1 and GPD1 is influenced by further factors and the increase in GPD1 expression is stronger than in Hog1 phosphorylation. Hence, we used Hill kinetics to describe the activation of GPD1 transcription. Since we have no knowledge on the nature of $AOG2$, we also used Hill kinetics for the second term in v_{17}:

$$\begin{aligned} v_{17f}(t) = & \frac{k_{v17f.1} \cdot Hog1PP(t)^{k_{v17f.6}}}{k_{v17f.2} + Hog1PP(t)^{k_{v17f.6}}} && \text{[Hog1-dependent]} \\ & + \frac{k_{v17f.3} \cdot AOG2(t)^{k_{v17f.7}}}{k_{v17f.4} + AOG2(t)^{k_{v17f.7}}} && \text{[Hog1-independent]} \\ & + k_{v17f.5} && \text{[basal]}. \end{aligned} \tag{9}$$

Transcription of STL1 (v_{20}) is specified in the same manner with the exception that the Hog1-independent term is omitted.

Activation of Pfk26/27 (v_{19}) is defined by modified mass action kinetics to minimize additional parameters in the description of this experimentally sparsely characterized reaction.

Changes in the abundance of open Fps1 (v_{22}) are stated as an equilibrium between positive and negative terms, where the opening terms are negatively affected by turgor and Hog1PP. Turgor-mediated closure of Fps1 is widely accepted in literature (Klipp et al., 2005, Mettetal et al., 2008) and the Hog1PP dependent term is included based on our findings as described in the main text.

Biomass production (v_{14}) was initially included into the model to allow for steady intracellular metabolite concentrations before stress and in unstressed models because the inflow of glucose exceeds the efflux of trehalose, acetate, ethanol and glycerol. To maintain a concise model, biomass production is described as one reaction branching off glycolysis from G6P. In order to reproduce experimentally observed glycerol accumulation as well as experimentally observed ethanol and acetate production in model simulations, biomass production must be rerouted to glycerol and pyruvate. Although Hog1 has been reported to inhibit cell cycle progression under hyperosmotic conditions (Escotet et al., 2004 and Clotet et al., 2006), we also observed diminished growth in *hog1*Δ experiments. We assume the decrease in biomass production to be influenced by volume-dependent signaling mechanisms and the cost of maintaining a high intracellular osmolarity in the face of sustained high salinity. Hence, v_{14} is described as Michaelis-Menten kinetics modified by cell volume Vos and external osmolarity $a_2(t)$.

Model Equations

Rate Equations for Biochemical Reactions

Differential equations as used in the model for WT. For parameter values and initial values, please refer to the SBML model in further Supplemental Information. All reaction rates are given in $\frac{\mathrm{M}}{\mathrm{s}}$

$$
\begin{aligned}
v_1(t) &= k_{v1.1} \cdot \frac{glc_e(t)/k_{v1.2}}{1 + glc_e(t)/k_{v1.2}} \\
v_2(t) &= k_{v2.1} \cdot \frac{glc_i(t)/k_{v2.2}}{1 + glc_i(t)/k_{v2.2}} \\
v_3(t) &= k_{v3.1} \cdot \frac{G6P(t)/k_{v3.2}}{1 + G6P(t)/k_{v3.2}} - k_{v3.4} \cdot \frac{tre_i(t)/k_{v3.4}}{1 + tre_i(t)/k_{v3.4}} \\
v_4(t) &= \left(k_{v4.2} \cdot \left(1 - \frac{F26DP(t)^{k_{v4.5}}}{(F26DP(t) + k_{v4.3})^{k_{v4.5}}}\right) + k_{v4.1} \cdot \frac{F26DP(t)^{k_{v4.5}}}{(F26DP(t) + k_{v4.3})^{k_{v4.5}}} \right) \\
&\quad \cdot \frac{(G6P(t)/k_{v4.4})^2}{1 + (G6P(t)/k_{v4.4})^2} \cdot \frac{|G6P(t)|}{G6P(t)} \\
v_5(t) &= k_{v5.1} \cdot \frac{F16DP(t)/k_{v5.2}}{1 + F16DP(t)/k_{v5.2}} - k_{v5.3} \cdot \frac{triose(t)/k_{v5.4}}{1 + triose(t)/k_{v5.4}} \\
v_6(t) &= k_{v6.1} \cdot Gpd1(t) \cdot \frac{triose(t)^{k_{v6.2}}}{k_{v6.3} + triose(t)^{k_{v6.2}}} \\
v_{6b}(t) &= \frac{k_{v6b.4} \cdot triose(t)^2/k_{v6b.5}}{1 + triose(t)^2/k_{v6b.5}} \\
v_7(t) &= k_{v7.1} \cdot \frac{triose(t)/k_{v7.2}}{1 + triose(t)/k_{v7.2}} \\
v_8(t) &= k_{v8.1} \cdot \frac{pyr(t)/k_{v8.2}}{1 + pyr(t)/k_{v8.2}} \\
v_9(t) &= k_{v9.1} \cdot \frac{pyr(t)/k_{v9.2}}{1 + pyr(t)/k_{v9.2}} \\
v_{10}(t) &= k_{v10.1} \cdot a_4(t) \cdot (tre_i(t) - k_{v10.2} \cdot tre_e(t)) \\
v_{11}(t) &= k_{v11.1} \cdot a_4(t) \cdot (ac_i(t) - k_{v11.2} \cdot ac_e(t)) \\
v_{12}(t) &= k_{v12.1} \cdot a_4(t) \cdot (EtOH_i(t) - k_{v12.2} \cdot EtOH_e(t)) \\
v_{13a}(t) &= Fps1r(t) \cdot k_{v13a.1} \cdot a_4(t) \cdot (glyc_i(t) - glyc_e(t)) \\
v_{13b}(t) &= k_{v13b.1} \cdot Stl1(t) \cdot \frac{glyc_e(t)}{k_{v13b.2} + glyc_e(t)} \\
v_{14}(t) &= \frac{\frac{k_{v14.1} V_{os}^{k_{v14.3}}}{V_{os}^{k_{v14.3}} + k_{v14.2}} \left(1 - \frac{a_2(t)}{a_2(t) + k_{v14.4}}\right) G6P(t)/k_{v14.5}}{1 + G6P(t)/k_{v14.5}} \\
v_{15f}(t) &= k_{v15f.1} \cdot Pfk2a(t) \cdot \frac{G6P(t)}{k_{v15f.2} + G6P(t)}
\end{aligned}
$$

$$v_{15r}(t) = k_{v15r.1} \cdot \frac{F26DP(t)}{k_{v15r.2} + F26DP(t)}$$

$$v_{16f}(t) = Hog1U(t) \cdot a_2(t) \cdot k_{v16f.1} \cdot \left(\frac{k_{v16f.2}}{a_1(t)}\right)^{k_{v16f.3}}$$

$$v_{16r}(t) = k_{v16r.1} \cdot Hog1P(t)$$

$$v_{17f}(t) = \frac{k_{v17f.1} \cdot Hog1P(t)^{k_{v17f.5}}}{Hog1P(t)^{k_{v17f.5}} + k_{v17f.2}} + \frac{k_{v17f.3} \cdot AOG(t)^{k_{v17f.6}}}{AOG(t)^{k_{v17f.6}} + k_{v17f.4}} + k_{v17f.7}$$

$$v_{17r}(t) = k_{v17r.1} \cdot gpd1m(t)$$

$$v_{18f}(t) = gpd1m(t) \cdot k_{v18f.1}$$

$$v_{18r}(t) = k_{v18r.1} \cdot Gpd1(t)$$

$$v_{19f}(t) = k_{v19f.1} \cdot Hog1P(t) \cdot Pfk2i(t)$$

$$v_{19r}(t) = k_{v19r.1} \cdot Pfk2a(t)$$

$$v_{20f}(t) = \frac{k_{v20f.1} \cdot Hog1P(t)^{k_{v20f.x}}}{Hog1P(t)^{k_{v20f.x}} + k_{v20f.2}} + k_{v20f.3}$$

$$v_{20r}(t) = k_{v20r.1} \cdot stl1m(t)$$

$$v_{21r}(t) = k_{v21r.1} \cdot Stl1(t)$$

$$v_{21f}(t) = stl1m(t) \cdot k_{v21f.1}$$

$$v_{22}(t) = \frac{k_{v22.1} \cdot (-a_3(t))}{k_{v22.3} + (-a_3(t))} \cdot \left(1 - \frac{Hog1P(t)}{Hog1P(t) + k_{v22.2}}\right) - k_{v22.1} \cdot Fps1r(t)$$

$$v_{AOG2r}(t) = k_{vAOG2r.1} \cdot AOG2a(t)$$

$$v_{AOG2f}(t) = AOG2i(t) \cdot k_{vAOG2f.2} \cdot \left(\frac{k_{vAOG2f.1}}{a[4]}\right)^{k_{vAOG2f.3}}$$

Rate Equations to Account for Biophysical Changes

Rates of change of V_{os} due to osmotic activity inside and outside the cell and changes in intracellular concentrations due to volume changes are computed as described here.

$$v_{V_{os}}(t) = k_{vV.1} a_4(t) \cdot \Big(a_3(t) - k_{vV.2} RT\Big(glyc_e(t) + a_2(t) - (glyc_i(t)) - Osmo_i(t)\Big)\Big)$$

$$v_{V\,species}(t) = species(t) \cdot \frac{v_{V_{os}}(t)}{V_{os}(t)}$$

Where $v_{V\,species}(t)$ indicates the volume-dependent change of intracellular concentration and is computed for each intracellular concentration as indicated in the differential equations.

Differential Equations

The rate equations given above are used to compute the changes in concentrations according to the following differential equations.

$$\frac{\mathrm{d}glc_e}{\mathrm{d}t} = -v_1(t)/c \cdot \frac{a_5(t)}{k_{batch}}$$
$$\frac{\mathrm{d}glc_i}{\mathrm{d}t} = v_1(t) - v_2(t) - v_{Vglyc_i}(t)$$
$$\frac{\mathrm{d}G6P}{\mathrm{d}t} = v_2(t) - 2v_3(t) - v_{14}(t) - v_{15f}(t) + v_{15r}(t) - v_{VG6P}(t)$$
$$\frac{\mathrm{d}tre_i}{\mathrm{d}t} = v_3(t) - v_{10}(t) - v_{Vtre_i}(t)$$
$$\frac{\mathrm{d}F16DP}{\mathrm{d}t} = v_4(t) - v_5(t) - v_{VF16DP}$$
$$\frac{\mathrm{d}F26DP}{\mathrm{d}t} = v_{15f}(t) - v_{15r}(t)$$
$$\frac{\mathrm{d}triose}{\mathrm{d}t} = 2v_5(t) - v_6(t) - v_{6b}(t) - v_7(t) - v_{Vtriose}(t)$$
$$\frac{\mathrm{d}glyc_i}{\mathrm{d}t} = v_6(t) + v_{6b}(t) - v_{13a} + v_{13b}(t) - v_{Vglyc_i}(t)$$
$$\frac{\mathrm{d}pyr}{\mathrm{d}t} = v_7(t) - v_8(t) - v_9(t) - v_{Vpyr}(t)$$
$$\frac{\mathrm{d}ac_i}{\mathrm{d}t} = v_8(t) - v_{11}(t) - v_{Vac_i}(t)$$
$$\frac{\mathrm{d}EtOH_i}{\mathrm{d}t} = v_9(t) - v_{12}(t) - v_{VEtOH_i}(t)$$
$$\frac{\mathrm{d}tre_e}{\mathrm{d}t} = v_{10}(t)/V_{extra} \cdot \frac{a_5(t)}{k_{batch}}$$
$$\frac{\mathrm{d}glyc_e}{\mathrm{d}t} = \frac{a_5(t)}{k_{batch}} \cdot (v_{13a}(t)/V_{extra} - v_{13b}(t)/V_{extra})$$
$$\frac{\mathrm{d}ac_e}{\mathrm{d}t} = v_{11}(t)/V_{extra} \cdot \frac{a_5(t)}{k_{batch}}$$
$$\frac{\mathrm{d}EtOH_e}{\mathrm{d}t} = v_{12}(t)/V_{extra} \cdot \frac{a_5(t)}{k_{batch}}$$
$$\frac{\mathrm{d}BM}{\mathrm{d}t} = v_{14}(t)$$
$$\frac{\mathrm{d}Hog1P}{\mathrm{d}t} = v_{16f}(t) - v_{16r}(t) - v_{VHog1P}(t)$$
$$\frac{\mathrm{d}Hog1U}{\mathrm{d}t} = -v_{16f}(t) + v_{16r}(t) - v_{VHog1U}(t)$$
$$\frac{\mathrm{d}gpd1m}{\mathrm{d}t} = v_{17f}(t) - v_{17r}(t) - v_{Vgpd1m}(t)$$
$$\frac{\mathrm{d}Gpd1}{\mathrm{d}t} = v_{18f}(t) - v_{18r}(t) - v_{VGpd1}(t)$$

$$\frac{\mathrm{d}stl1m}{\mathrm{d}t} = v_{20f}(t) - v_{20r}(t) - v_{Vstl1m}(t)$$
$$\frac{\mathrm{d}Stl1}{\mathrm{d}t} = v_{21f}(t) - v_{21r}(t) - v_{VStl1}(t)$$
$$\frac{\mathrm{d}Fps1r}{\mathrm{d}t} = v_{22}(t)$$
$$\frac{\mathrm{d}Pfk2a}{\mathrm{d}t} = v_{19f}(t) - v_{19r}(t) - v_{Pfk2a}(t)$$
$$\frac{\mathrm{d}Pfk2i}{\mathrm{d}t} = -v_{19f}(t) + v_{19r}(t) - v_{VPfk2i}(t)$$
$$\frac{\mathrm{d}V_{os}}{\mathrm{d}t} = v_{V_{os}}(t)$$
$$\frac{\mathrm{d}Osmo_i}{\mathrm{d}t} = -v_{VOsmo_i}(t)$$
$$\frac{\mathrm{d}AOG2a}{\mathrm{d}t} = v_{AOG2f}(t) - v_{AOG2r}(t)$$
$$\frac{\mathrm{d}AOG2i}{\mathrm{d}t} = -v_{AOG2r}(t) + v_{AOG2r}(t)$$

Algebraic equations

Algebraic equations used in the wild-type model ($a_6(t)$ is a fit to experimental data for each strain):

V_m	$a_1(t) = V_b + V_{os}(t)$
$Osmo_e$	$a_2(t) = Osmo_e(0) + u_1(t) \cdot 0.8$
$Turgor$	$a_3(t) = \begin{cases} a_3(0) \cdot \left(1 - \frac{V_{os}(0) - V_{os}(t)}{V_{os}(0) - V_{a_3=0}}\right) & \text{if } V_{os}(t) > V_{a_3=0} \\ 0 & \text{else} \end{cases}$
$CellSurface$	$a_4(t) = (36.0 \cdot \pi)^{1/3} \cdot a_1(t)^{2/3}$
$celldensity$	$a_5(t) = -6548240 \cdot a_6(t)^2 + 30565100 \cdot a_6(t) - 4727510$
OD	$a_6(t) = 2.94557 \times 10^{-9} t^2 + 6.49182 \times 10^{-5} t + 0.595608$

Experimental data for intracellular concentrations was processed assuming a constant volume. Hence, algebraic equations are used to compute intracellular concentrations without the effect of volume changes to compare with experimental data:

$species_{NoVol}$	$a_{species}(t) = species(t) \cdot V_{os}(t)/V_{os}(0)$

For all intracellular species ($Hog1U$, $Hog1P$, $Gpd1$, $gpd1m$, glc_i, pyr, ac_i, $EtOH_i$, tre_i, $F16DP$, $triose$, $G6P$, $stl1m$, $Stl1$, $glyc_i$).

Salt stress is introduced into the model assuming a mixing time of 5 seconds from the onset of the stress at t_s:

$stress$	$u_1(t) = \begin{cases} 0 & \text{if } t < t_s, \\ (t - t_s)/5 & \text{if } t_s \le t \le t_s + 5 \\ 1 & \text{else} \end{cases}$

Model Variants for Mutant Strains

Besides initial conditions and the function to reproduce experimentally determined OD according to the respective experimental data, the models for each mutant strain had to be modified to reproduce the biological changes with respect to osmoadaptation.

Wherever possible, we applied minimalistic modification not taking into account possible wider effects of the mutations on cellular state. Full models are given as additional Supplemental Material.

Changes for *FPS1*-Δ*1*

- Initial concentration of $Fps1$ is increased/

- Reaction $v_{22}(t)$ is set to 0.
- Reaction $v_{16r}(t)$ contains a time-dependent parameter to force a decrease in Hog1 activity after $t = 4800s$ as observed in experimental data.

The variable parameter in $v_{16r}(t)$ is necessary because Hog1 activity in our model would only diminish if volume is regained. Physiologically, Hog1 activity is not only controlled by volume but also by feedback through phosphatases, which is included via this modification.

Changes for *gpd1*Δ

- Initial concentrations for $Gpd1$ and $gpd1mRNA$ are set to 0.
- Reaction $v_6(t)$ is set to 0.
- Reaction $v_{17f}(t)$ is set to 0.

Changes for *HOG1-att*

- The first, Hog1-dependent part of reaction $v_{17f}(t)$ is set to 0, only Hog1-independent and basal transcription remains.
- The first, Hog1-dependent part of reaction $v_{20f}(t)$ is set to 0, only basal transcription remains.
- Reaction $v_{22}(t)$ is modified to account for the higher interaction of membrane-tethered Hog1 with Fps1:

$$\begin{aligned} v_{22}(t) =& k_{v22.1} \cdot 1.5 \cdot \frac{|a_3(t)|}{k_{v22.2} + |a_3(t)|} \\ & \cdot (1.0 - \frac{Hog1PP(t) \cdot 1.29462}{(Hog1PP(t) \cdot 1.29462 + k_{v22.3}}) \\ & - k_{v22.1} \cdot Fps1r(t). \end{aligned}$$

Changes for *hog1*Δ

- Hog1 and Hog1PP initial concentrations set to 0.
- $v_{16f}(t)$ and $v_{16r}(t)$ set to 0.
- $v_{19f}(t)$ and $v_{19r}(t)$ set to 0.
- $v_{22}(t)$ contains a time-dependent parameter that forces reopening of Fps1 after $t = 5536s$ to reproduce increase in extracellular glycerol as observed in experiments.

The time-dependent parameter in $v_{22}(t)$ does not give a mechanistic explanation why Fps1 reopens (in our opinion the only sensible reason for an increase in extracellular glycerol as observed in experiments) while Turgor is not regained, but is necessary to reproduce experimental data.

Changes to *pfk26/27*Δ

- Initial concentrations of $Pfk26/27a$ and $Pfk2627i$ set to 0.
- To avoid numerical issues, reactions $v_{19f}(t)$, $v_{19r}(t)$, $v_{15f}(t)$, and $v_{15r}(t)$ were set to 0.

Model Simulations

Simulation of additional model variables not presented in the main text are given in Supplementary Figure S12, S13 and S14.

4 Parameter Estimation Details

Comparison to Processed Experimental Data

Experimental data was processed assuming a constant cell volume (see above). The model contains changes of cellular volume which affect intracellular concentrations. The simulated volume-sensitive intracellular concentrations $C_{intra}(t)$ are used in model equations. For parameter estimation, variables that calculate volume-insensitive intracellular concentrations $C_{measured}(t)$ are used:

$$C_{measured}(t) = C_{intra}(t) \cdot \frac{Vos(t)}{Vos(0)} \tag{10}$$

In order to reduce the parameter boundaries in parameter estimation, some reactions parameters were estimated individually with Copasi. This is only possible for reactions for which all reactants, products and modifiers are well determined in experimental data. Here, this is the case for reactions v_1 and $v_{16f/r}$, for example. The resulting estimates have been used to guide subsequent large scale parameter estimation.

Estimation Methods

We constructed models for wild-type, *FPS1-Δ1*, *gpd1*Δ, *HOG1-att*, *hog1*Δ and *pfk26/27*Δ. Initially, each model was fitted independently to the corresponding experimental data. Simulating one model with the parameter set (excluding initial concentrations) of another (e.g. the wild-type model with parameters from the *HOG1-att* model) did not yield a satisfactory agreement with experimental data.

To generate one parameter set that suits all models, we combined all models and datasets in PottersWheel and estimated parameters using Simulated annealing and TrustRegion in linear and logarithmic parameter space. The resulting fits were analyzed and fitting was refined:

1. In order to improve the goodness of fits, trehalose was excluded from parameter estimation because the model contains no regulation of trehalose production, consumption and transport and can hence not be expected to reproduce trehalose dynamics.

2. In order to improve the goodness of fits concerning reproduction of adaptation dynamics, noisy metabolite data (e.g. intracellular ethanol for WT4) was removed.

3. In order to reduce computational costs and numerical difficulties, the set of parameters to be estimated was reduced iteratively. This is especially relevant for parameters used as exponents (e.g. $k_{v16f.3}$).

Estimation Results

In the series of fits that includes the parameter set selected for the main text, we fitted 87 parameters, including 4 to describe additional effects in the *hog1*Δ and *HOG1-att* strains, to a total of 995 data points. Of 308 fitting runs, at least 105 did not produce usable results due to numerical errors (for some of the models, integration fails, no time course is produced and an erroneous, small χ^2 is reported). The parameter set used for simulations has the lowest χ^2-value without numerical errors: $\chi^2 = 643.274$.

The high χ^2 can be largely attributed to differences between simulation and experimental data for $FPS1$-Δ1 and to experimental data for intracellular acetate, ethanol,glucose and pyruvate where the overall shape of the experimental data is reproduced well but individual measurement errors contribute significant errors. Additionally, the time course for most intracellular metabolites is smooth compared to the large osmoadaptation-dependent changes in, for example, intracellular glycerol and Hog1PP.

The 10 best fits with χ^2 between 619.34 and 656.082 were found a total 15 times, the best fit was found 4 times. These best fits are very similar considering χ^2 error, even of individual model variables, and parameter values. Comparison of the 10 best fits thus indicates that fitting multiple strains greatly reduces the number of possible parameter sets because the data for each strain must be reproduced.

5 Modifications to improve model of *FPS1-Δ1*

All genetic perturbations were implemented including all their effects to the best of our knowledge. However, genetic perturbations inadvertandly lead to an adaptation of the cellular state to cope with the effect of the perturbation. This can in turn generate indirect effcts that need to be accounted for in strains where the general state is significantly affected by the genetic perturbation. Additionally, a perturbation in fps1 can induce changes in dynamics of Hog1 if the interaction between Hog1 and Fps1 suggested in this work is true.

The following suggestion is highly speculative and presents just one possibility out of potentially many to improve the model.

The fit to data from *FPS1*-Δ*1* can be improved by changing parameter values. These deviations from the general parametrization can be interpreted as modifications to the cellular state that *FPS1*-Δ*1* imposes on the cells. We have found one set of such modifications which improves the fit to experimental data, see Supplemental Figure S16. The changes applied are:

- decrease gpd1 transcription by a factor of 10,
- decrease gpd1mRNA degradation by a factor of 80,
- decrease Gpd1 degradation by a factor of 128.

the first modification could be attributed to a slower translocation to the nucleus of Hog1, the latter two modifications could be the results of cellular adaptation to *FPS1*-Δ*1* to increase gpd1 production in the unstressed state already. However, this parametrization results in a Gpd1 concentration up to 6 times higher than measured in *FPS1*-Δ*1* and reproduced well with the general parametrization (see Figure S13C). Therefor, it seems as if *FPS1*-Δ*1* cells likely increase glycerol production not by an increase in Gpd1 but by an alternative route, e.g. Gpd2.

6 Response Coefficients

Scaled time-dependent response coefficients (RCs) have been computed for the model as described in main text (mathematica code for computation is available upon request). Response coefficients describe the effect of small variations in one parameter on a certain concentration. Analysis of response coefficients is demanding for a model of the size used here (6 models with about 90 parameters and 25 model variables) because of the number of response coefficients (see Supplemental Figure S15 for an extended selection of response coefficients). Two pitfalls for the analysis of response coefficients must be noted:

- RCs measure the effect of a change in a single parameter. Hence, RC analysis likely does not match observed biological perturbation effects quantitatively because living cells usually adjust to a perturbation (as shown in this manuscript). Adjusting to the perturbation in a single parameter usually involves changing additional parameters. This is especially true for perturbations in glycolytic pathways, e.g. the effect of *pfk26/27*Δ and the corresponding RC depicted in Figure S15.
- RCs depend on the usage of the parameter inside a rate law. A negative RC for a K_m value would indicate an increase in species concentration when the K_m-value is increased. Likewise, a single RC does not necessarily reflect the influence of a reaction.
- RCs reflect the effect of small changes in parameter values. For variables with a switch-like behavior (e.g. Hog1 and Fps1 in our model, compare Supplemental Figure S15), a small change in a corresponding parameter will not affect the variables state and hence not influence the overall system.

RCs can visualize the dramatic changes in the contribution of different mechanisms to an overall effect, as exemplified in Supplemental Figure S15B, E, F: One part of the changes in response is due to the direct effects of the perturbations, but the system passively adjusts to the perturbation and a different mechanisms contribution to the overall process is increased.
